# A regulatory variant at *3q21.1* confers an increased pleiotropic risk for hyperglycemia and altered bone mineral density

**DOI:** 10.1016/j.cmet.2021.01.001

**Published:** 2021-03-02

**Authors:** Nasa Sinnott-Armstrong, Isabel S. Sousa, Samantha Laber, Elizabeth Rendina-Ruedy, Simon E. Nitter Dankel, Teresa Ferreira, Gunnar Mellgren, David Karasik, Manuel Rivas, Jonathan Pritchard, Anyonya R. Guntur, Roger D. Cox, Cecilia M. Lindgren, Hans Hauner, Richard Sallari, Clifford J. Rosen, Yi-Hsiang Hsu, Eric S. Lander, Douglas P. Kiel, Melina Claussnitzer

**Affiliations:** 1 Metabolism Program, Broad Institute of MIT and Harvard, Cambridge, MA 02142, USA; 2 Program in Medical and Population Genetics, Broad Institute of MIT and Harvard, Cambridge, MA 02142, USA; 3 Cell Circuits and Epigenomics, Broad Institute of MIT and Harvard, Cambridge, MA 02142, USA; 4 Department of Genetics, Stanford University, Stanford 94305 CA, USA; 5 Else Kröner-Fresenius-Center for Nutritional Medicine, School of Life Sciences, Technical University of Munich, Freising 85354, Germany; 6 Big Data Institute, University of Oxford, Oxford, UK; 7 Center for Molecular Medicine, Maine Medical Center Research Institute, Scarborough, ME 04074, USA; 8 University of Bergen, Bergen 5020, Norway; 9 Mohn Nutrition Research Laboratory, Department of Clinical Science, University of Bergen, 5020 Bergen, Norway; 10 Hormone Laboratory, Department of Medical Biochemistry and Pharmacology, Haukeland University Hospital, 5021 Bergen, Norway; 11 Institute for Aging Research, Hebrew SeniorLife and Harvard Medical School, Boston, MA 02131, USA; 12 Faculty of Medicine of the Galilee, Bar-Ilan University, Safed, Israel; 13 Department of Biomedical Data Science, Stanford University, Stanford, CA 94305, USA; 14 Department of Biology, Stanford University, Stanford, CA 94305, USA; 15 Institute of Nutritional Medicine, School of Medicine, Technical University of Munich, Freising 85354, Germany; 16 Clinical Cooperation Group “Nutrigenomics and Type 2 Diabetes” of the German Center of Diabetes Research, Helmholtz Center Munich, Munich 85764, Germany; 17 Department of Medicine, Beth Israel Deaconess Medical Center, Harvard Medical School, Boston, MA 02131, USA; 18 Department of Biology, MIT, Cambridge, MA 02142, USA; 19 Department of Systems Biology, Harvard Medical School, Boston, MA 02115, USA; 20 Medical Research Council Harwell, Oxfordshire, UK; 21 University of Hohenheim, Institute of Nutritional Science, Stuttgart 70599, Germany

**Keywords:** variant-to-function study, pleiotropy of type 2 diabetes and bone mineral density, osteoblast and adipocyte metabolism, regulatory genomics, CRISPR-Cas9 variant editing

## Abstract

Skeletal and glycemic traits have shared etiology, but the underlying genetic factors remain largely unknown. To identify genetic loci that may have pleiotropic effects, we studied Genome-wide association studies (GWASs) for bone mineral density and glycemic traits and identified a bivariate risk locus at *3q21*. Using sequence and epigenetic modeling, we prioritized an adenylate cyclase 5 (ADCY5) intronic causal variant, rs56371916. This SNP changes the binding affinity of SREBP1 and leads to differential *ADCY5* gene expression, altering the chromatin landscape from poised to repressed. These alterations result in bone- and type 2 diabetes-relevant cell-autonomous changes in lipid metabolism in osteoblasts and adipocytes. We validated our findings by directly manipulating the regulator SREBP1, the target gene *ADCY5*, and the variant rs56371916, which together imply a novel link between fatty acid oxidation and osteoblast differentiation. Our work, by systematic functional dissection of pleiotropic GWAS loci, represents a framework to uncover biological mechanisms affecting pleiotropic traits.

## Highlights

• *3q21* variants are associated with type 2 diabetes and increased bone mineral density
• *3q21* variants affect chromatin accessibility in mesenchymal cells
• rs56371916 is a *3q21* causal variant and *ADCY5* its target in adipocytes and osteoblasts
• *ADCY5* and rs56371916 affect lipid oxidation processes in adipocytes and osteoblasts

## Graphical abstract

**Figure:**
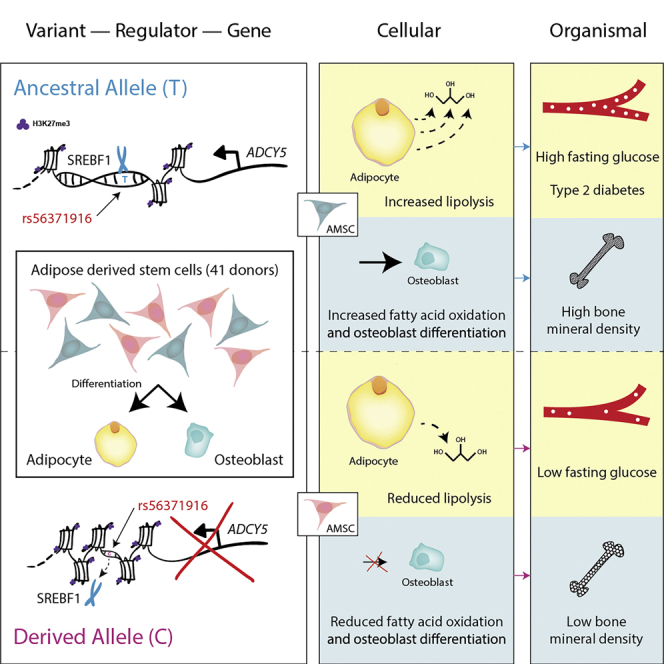

## Teaser

Nasa Sinnott-Armstrong and colleagues identify a pleiotropic risk locus at *3q21* that is associated with type 2 diabetes (T2D) and greater bone mineral density (BMD) and its associated cell-autonomous mechanisms in adipocytes and osteoblasts. Together, these findings provide a possible explanation for the perplexing finding that individuals with T2D have higher BMD but greater susceptibility to bone fracture.

## Introduction

Patients with type 2 diabetes (T2D) have increased bone mineral density (BMD) yet greater susceptibility to fracture (Vestergaard, 2007). This perplexing finding suggests an intimate link between skeletal and metabolic traits. While some have suggested that this observation implies shared genetic etiology between skeletal and metabolic traits in humans (Vestergaard, 2007; Billings et al., 2012) the connection between the molecular and cellular mechanisms underlying T2D and BMD remain unknown, and no systematic studies of their shared genetics have been published, to the best of our knowledge. Thus, we studied BMD and glycemic traits as quantitative phenotypes of the skeleton and T2D, respectively, as both traits have high heritability and polygenicity. We hypothesized that large-effect genetic variants that alter both bone and glycemic traits would elucidate the mechanism by which the shared etiology of T2D and increased BMD (but more fragile bones) occurs. Genome-wide association studies (GWASs) have identified tens of thousands of genomic loci underlying individual human traits, including BMD and glycemic traits, of which the latter consists of fasting glucose, fasting insulin, HOMA-IR, and HOMA-B. However, these GWAS-identified loci have only rarely been resolved into causal variants or resulted in enumerated underlying mechanisms due to several challenges (Eichler et al., 2010). The vast majority of loci involve non-coding variants that likely act through regulatory changes; over 80% of GWAS-identified loci contain no protein-altering common variants, even when considering all variants in linkage disequilibrium (LD) at R^2^ ≥ 0.8 (Hindorff et al., 2009), making it difficult to pinpoint the causal variants, regulatory circuits, relevant cell types and tissues, key developmental stages, and affected cellular functions (Cai et al., 2003; Claussnitzer et al., 2015; Steidl et al., 2007; Cowper-Sal lari et al., 2012). Moreover, there is growing evidence of pervasive pleiotropy, with single genetic variants affecting two or more seemingly unrelated traits (Sivakumaran et al., 2011). In fact, hundreds of individual variants identified from GWASs are associated with multiple traits (Bulik-Sullivan et al., 2015), with effects in multiple cell types. Thus, new approaches to dissect genetic risk loci are desperately needed. A systematic study of pleiotropic loci represents an opportunity to discover biological mechanisms underlying the individual traits and further mechanisms that link these traits.

Here, we use GWAS summary statistics to identify genetic loci that may have pleiotropic effects on skeletal and glycemic traits. We used femoral neck BMD and lumbar spine BMD as quantitative endophenotypes that are strongly predictive of osteoporotic fracture, and fasting glucose, fasting insulin, HOMA-IR, and HOMA-B to define T2D. We elucidated the functional basis of the most intriguing bivariate GWAS signal, a locus at *3q21.1*, which was associated with femoral neck BMD and fasting glucose. We discovered that the GWAS signal was driven by rs56371916, an intronic variant in adenylate cyclase 5 (*ADCY5*) that alters the binding affinity of sterol regulatory-element-binding protein 1 (SREBP1) and leads to differential *ADCY5* gene expression and cell-autonomous changes in fatty acid metabolism in mature adipocytes and differentiating osteoblasts. Importantly, we demonstrate that disruption of each, the regulator SREBP1, the variant rs56371916, and the target gene *ADCY5* cause cellular changes (e.g., lipid oxidation) relevant to BMD and T2D. Together, our work identifies a novel link between fatty acid oxidation and osteoblast differentiation. But more generally, by leveraging the identification and functional dissection of pleiotropic GWAS loci, we introduce a framework to uncover novel biological mechanisms affecting more than one disease trait.

## Results

### GWAS identifies bivariate loci for BMD and glycemic traits, including a locus at *3q21.1*

To discover genetic loci with possible pleiotropic effects on glycemic traits and BMD, we used GWAS summary statistics from the MAGIC consortium (in which the four glycemic traits HOMA-IR, HOMA-B, fasting glucose levels, and fasting insulin levels were measured) (Dupuis et al., 2010; Manning et al., 2012) and the GEFOS consortium (in which femoral neck BMD [FNBMD] and lumbar spine BMD [LSBMD] were measured) (Estrada et al., 2012).

To identify candidate pleiotropic loci, we used the CP-ASSOC program (Park et al., 2016) to consider all 8 pairs of the two BMD traits and four glycemic traits. We identified 8 distinct bivariate loci; that is, loci effects on both BMD and glycemia (bivariate p ≤ 5 × 10^−6^) (STAR methods; Table 1; Figure S1A). Consistent with most published GWASs, only one locus (at *GCKR*) harbored a protein-coding variant in strong LD with the lead variant. Notably, heritability partitioning across the entire bivariate GWAS revealed that the bivariate signal was globally enriched for enhancer annotations, particularly for H3K4me1 and H3K27ac marks, signifying active and primed enhancers, in the mesenchymal lineage, including adipocytes, osteoblasts, and other mesenchymal cells (Figures S1B and S1C) (Finucane et al., 2015).

**Table 1:** Independent loci implicated by CP-ASSOC for association with both BMD and glycemic traits

Method	Locus	SNP rs#	Chr	Pos (GRCh37)	Ancestral	Derived	Derived Allele Frequency	Variant Classes	Bivariate	Bone Traits (GEFOS)			Glycemic Traits (MAGIC)		
									P-value	Trait	P-value	beta	Trait	P-value	beta
**CP-ASSOC**	*GCKR*	rs780110	2	27685388	A	G	0.56	3’UTR, synonymous	2.54E-15	LSBMD	4.49E-05	4.08	FG	2.84E-12	6.99
		rs1260326	2	27730940	C	T	0.40	missense	1.44E-10	LSBMD	3.64E-03	2.91	FI	1.18E-08	5.70
	*IGF1*	rs2607988	12	102929883	G	A	0.84	intergenic	4.71E-11	LSBMD	7.15E-03	-2.69	FI	1.96E-09	-6.00
	*ADRA2A*	rs11595612	10	112972505	C	T	0.09	intergenic	1.03E-10	LSBMD	5.29E-03	2.79	FG	1.07E-09	6.10
		rs11195496	10	113021531	G	T	0.09	intergenic	6.22E-09	FNBMD	6.78E-03	2.71	HOMAB	2.47E-07	5.16
	*TCF7L2*	rs17747324	10	114752503	T	C	0.22	intronic	9.14E-09	FNBMD	9.57E-03	-2.59	HOMAB	2.33E-07	-5.17
	*CYP19A1*	rs1062033	15	51547938	C	G	0.46	intronic	1.11E-07	FNBMD	1.61E-05	-4.31	HOMAIR	1.32E-04	-3.82
								intronic	4.20E-07	FNBMD	1.61E-05	-4.31	HOMAB	1.45E-03	-3.19
	*ADCY5*	rs2124500	3	123093530	C	T	0.27	intronic	1.83E-07	FNBMD	2.96E-03	2.97	FG	2.26E-06	4.73
		rs11717195	3	123082398	T	C	0.25	intronic	9.25E-07	FNBMD	3.99E-03	2.88	HOMAB	5.39E-05	4.04
	*POM121C*	rs6944634	7	75061769	C	G	0.19	intronic	2.46E-07	LSBMD	3.22E-03	-2.95	FI	1.23E-05	-4.37
	*SUSD4*	rs17161988	1	223444263	A	G	0.57	intronic	2.82E-07	FNBMD	6.54E-05	3.99	HOMAB	4.71E-04	3.50

**Figure 1:**
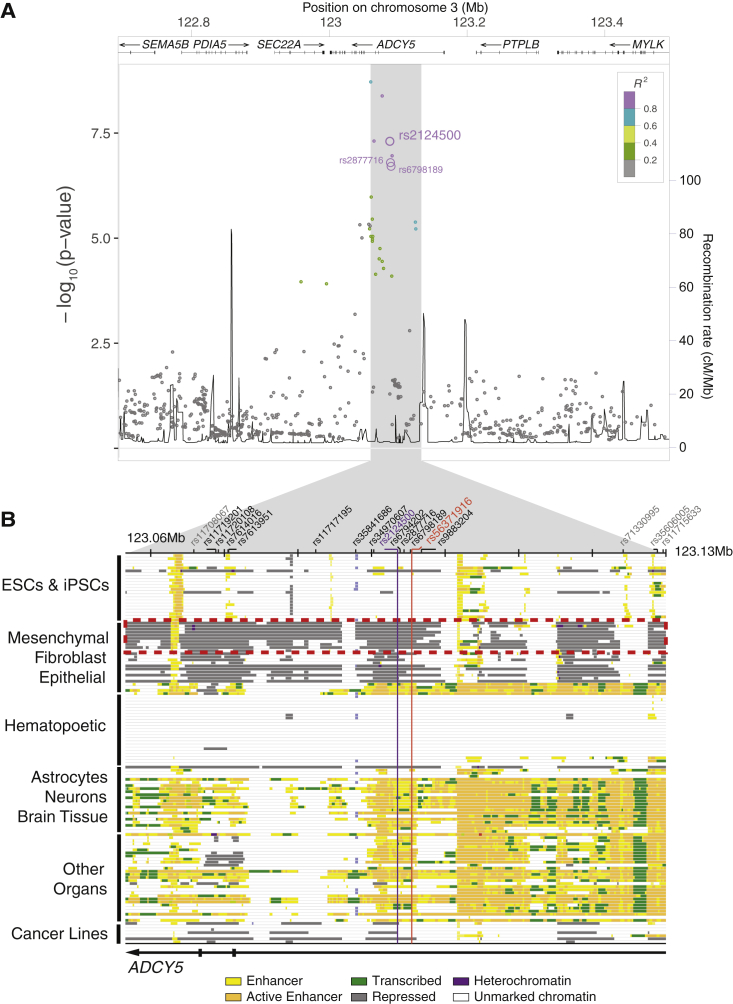
Pleiotropic *3q21.1* risk locus association with bone mineral density and fasting glucose levels (A) Bivariate genetic association with FNBMD and fasting glucose levels for *3p21.1* locus variants. LD in Europeans with the bivariate tag SNP rs2124500 is indicated (1,000 genomes R^2^, dot color). SNPs that meet bivariate criteria are marked by bolded purple dots. (B) Chromatin state annotations for the 65-kb-long *3p21.1* risk locus across 127 human cell types and tissues (Roadmap Epigenomics Consortium et al., 2015). Chromatin states include Polycomb-repressed states (gray), weak enhancers (yellow), strong enhancers (orange), and transcribed enhancers (lime).

A particularly interesting bivariate locus occurred at *3q21.1*, within an intron of the *ADCY5* gene, as the signal was seen across multiple traits, methods, and datasets. The locus was associated bivariately with FNBMD and glucose levels (lead SNP rs2124500, bivariate GWAS p = 1.83 × 10^−7^, Figure 1A), as well as individually with both FNBMD and HOMA-B (Tables 1 and S1). The locus also showed a bivariate association when tested with two other established methods: multi-trait analysis of GWAS (MTAG) (Turley et al., 2017) (p = 3.22 × 10^−9^) and eLX (Chen and Hsu, 2017) (p = 5.73 × 10^−9^) (Table S1). Additionally, the locus showed bivariate association (CP-ASSOC p = 1.35 × 10^−7^; eLX p = 4.35 × 10^−11^; MTAG p = 6.03 × 10^−9^) in independent data from the UK Biobank for heel BMD (n = 194,398) and diagnosed diabetes (n = 336,473) (Table S1). Finally, the *3q21.1* locus has recently been associated with greater heel BMD (UK Biobank [Morris et al., 2019], beta = 0.012, p = 7.6 × 10^−10^, n = 435,039), T2D (DIAMANTE [Mahajan et al., 2018], OR = 1.08, p = 6.5 × 10^−27^, n = 898,130), and nominally with lower fracture risk (Trajanoska et al., 2018), OR = 0.97, p = 1.7 × 10^−3^, n = 264,973) (Table 1).

### Haplotypes at *3q21.1* differ in chromatin accessibility and regulatory activity

The *3q21.1* locus is contained entirely within the 95-kb-long first intron of *ADCY5*, spanning 65 kb and harboring a set of 13 non-coding SNPs in strong LD (R^2^ > 0.8, 1000G Phase 1 EUR) (Figure 1A). These 13 variants (referred to here as the candidate regulatory variants) define two alternative haplotypes: the ancestral haplotype 1 (frequency 77% in European individuals), associated with higher FNBMD and higher fasting glucose levels, and haplotype 2 (frequency 23%), associated with lower FNBMD and lower fasting glucose levels.

**Figure 2:**
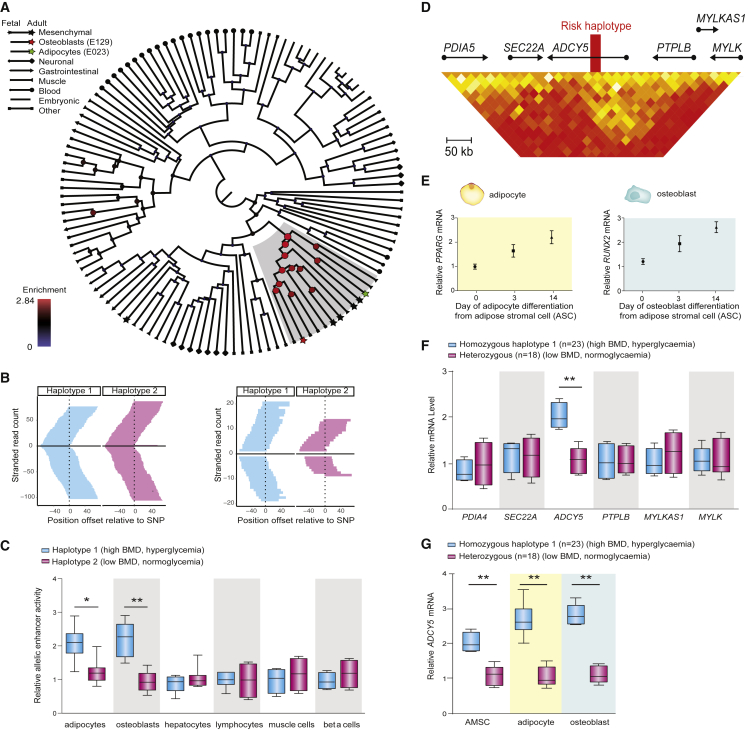
*ADCY5* expression differences between haplotypes in adipocytes and osteoblasts (A) H3K27me3 enrichment at *3q21.1* across multiple cell types. Linkage cluster tree displays node sizes and colors proportional to the H3K27me3 enrichment. Mesenchymal cells, adipocytes, and osteoblasts are highlighted by colored stars. (B) Allelic imbalance analysis of the *3p21.1* risk locus for H3K27me3 and chromatin accessibility by ATAC-seq in heterozygous AMSCs comparing haplotype 1 (blue) and haplotype 2 (pink). Each panel depicts H3K27me3 ChIP-seq and ATAC-seq read counts separated by strand above/below the midpoint. (C) Haplotype-specific luciferase assays for 10-kb fragments containing 10 candidate regulatory SNPs from each haplotype in tight LD with rs2124500 (R^2^ > 0.9) in adipocytes, osteoblasts, hepatocytes, lymphocytes, differentiated muscle cells, and pancreatic beta cells. Mean ± SD, ^∗^p < 0.05, ^∗∗^p < 0.01. (D) Genome-wide higher order chromatin interactions for the *ADCY5* locus analyzed by Hi-C assays in human embryonic stem-cell-derived MSCs (Dixon et al., 2015). (E) Quantification of adipocyte (yellow) and osteoblast (blue) differentiation marker gene expression from qPCR analysis across the course of differentiation. Three biological replicates, mean ± SD. (F) Quantification of *3p21.1* risk haplotype-dependent candidate target gene expression from qPCR analysis in AMSC-derived osteoblasts from n = 18 haplotypes 1/2 carriers and n = 23 haplotypes 1/1 carriers. Mean ± SD, ^∗∗^p < 0.01. (G) Quantification of *3p21.1* risk haplotype-dependent *ADCY5* target gene expression from qPCR analysis in AMSCs, AMSC-derived osteoblasts and AMSC-derived adipocytes from n = 18 haplotypes 1/2 carriers and n = 23 haplotypes 1/1 carriers. Mean ±SD, ^∗∗^p < 0.01.

To identify the cell types in which the causal variant(s) may act, we further examined chromatin state maps of the *3q21.1* locus across 127 human cell types (Roadmap Epigenomics Consortium et al., 2015) (Figures 1B and S1D). The data revealed that the entire locus was spanned by Polycomb-repressed chromatin (marked by H3K27me3) in mesenchymal lineages, while it was unmarked or active in non-mesenchymal cell types. Among the mesenchymal lineages, we focused on adipocytes, osteoblasts, and mesenchymal stem cells (MSCs), (which can give rise to adipocytes, osteoblasts, myocytes, and chondrocytes [Figure S2A]); these cell types had among the highest levels of enrichment for Polycomb-repressed chromatin (Figure 2A).

We examined whether the two haplotypes show differences in chromatin structure during adipocyte differentiation. Specifically, we performed assays for Polycomb repression (H3K27me3 ChIP-seq), enhancer activity (H3K27ac ChIP-seq), and chromatin accessibility (ATAC-seq) on adipose-derived mesenchymal stem cells (AMSCs) from a heterozygous individual for the *3p21.1* risk locus across a time course of differentiation (before induction [day 0], early differentiation [day 2], and terminal differentiation [day14]) and compared the numbers of reads from the two haplotypes. The two haplotypes showed no significant differences with respect to Polycomb repression (Figure 2B, left), but a striking difference in chromatin accessibility, with haplotype 1 being enriched by roughly 1.9-fold at all time points (Figure 2B, right; Table S2). The two haplotypes showed low levels of H3K27 acetylation without evidence of allelic imbalance (data not shown). A similar increase in chromatin accessibility of haplotype 1 is also evident in a replication sample of ATAC-seq performed on differentiating AMSCs from two additional individuals (Figure S1E; combined imbalance across all three individuals 1.57-fold, p = 0.0002), as well as published DNaseI hypersensitivity sequencing (DHS-seq) data in skeletal muscle-derived MSCs (Figure S1F) (Maurano et al., 2015). Consistent with recent studies showing that Polycomb repression can co-occur with chromatin accessibility (Muerdter et al., 2018; Scharer et al., 2018), these results indicate that haplotype 1 is associated with a poised state, whereas haplotype 2 is associated with a repressed state.

Further, we functionally tested the two haplotypes for differences in regulatory activity, using plasmid-based luciferase reporter assays in osteoblasts and adipocytes. Analysis of a 10-kb region containing the 10 candidate regulatory SNPs in tight LD with rs2124500 (R^2^ > 0.9) showed that haplotype 1 had 1.9-fold and 1.8-fold greater transcriptional activity in osteoblasts and adipocytes, respectively. In contrast, we saw no haplotype-specific regulatory differences in hepatocytes, lymphocytes, differentiated muscle cells, or pancreatic beta cells (Figure 2C).

### Regulatory region at *3p21.1* targets *ADCY5*

To identify potential regulatory target(s) of the locus, we examined three-dimensional genome folding maps from Hi-C assays in embryonic stem-cell-derived MSCs (Dixon et al., 2015). The locus lies in a well-defined 300-kb contact domain containing only two genes: *ADCY5* and *PTPLB* (Figure 2D). In our assessment, we considered the six genes within a larger 1-Mb region centered on the locus (*PDIA5*, *SEC22A*, *ADCY5*, *PTPLB*, *MYLKAS1*, and *MYLK*).

We isolated AMSCs from 41 normal-weight individuals, comprising 18 heterozygous individuals (haplotypes 1/2) and 23 homozygous individuals (haplotypes 1/1) (Cohort 1, see STAR methods). These AMSCs were then differentiated into mature osteoblasts and adipocytes, as confirmed by marker gene expression, bright field microscopy, and colorimetric assays (Figure 2E; Table S3; Figure S2B). Among the six genes, only *ADCY5* showed haplotype-specific differences in gene expression (Figure 2F), with haplotype 1 (homozygous individuals) being associated with 2.7-fold higher expression compared with haplotype 1/2 (heterozygous individuals) in both adipocytes and osteoblasts (p = 0.007, Figure 2G). These results implicate *ADCY5* as the likely regulatory target of the *3q21.1* locus.

### Computational analysis implicates rs56371916 as the likely causal variant

**Figure 3:**
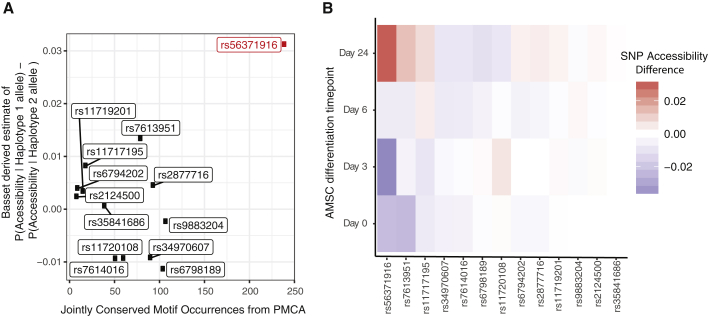
Sequence-based computational predictions of regulatory variants at the *3q21.1* locus (A) Phylogenetic conservation analysis (PMCA; Claussnitzer et al., 2014) across 20 vertebrate species and deep CNN-based predictions of chromatin accessibility for 13 highly linked SNPs at the *3q21.1* locus. (B) CNN-based Basset predictions of chromatin accessibility for *3q21.1* risk locus variants across a time course of differentiation. Data represent SNP accessibility difference scores at four stages of differentiation.

We next sought to identify which of the 13 candidate regulatory variants was likely to be responsible for the differential expression of *ADCY5*. We used two orthogonal computational approaches to prioritize variants and found that both highlighted the same SNP; namely, rs56371916. The first method, phylogenetic module complexity analysis (PMCA) (Claussnitzer et al., 2014, 2015; Hindorff et al., 2009) groups at least three transcription-factor-binding motifs within a 120-bp-region that show good evolutionary conservation of sequence, order, and distance (in human and at least one other vertebrate species). One variant, rs56371916, stood out as showing the highest score (Figure 3A; Table S4). The second method, Basset (Kelley et al., 2016), uses a sequence-based deep convolutional neural network (CNN) approach to predict effects of non-coding variants, by training on the sequence content of genomic regions strongly enriched for a given epigenomic mark in a tissue or cell type of interest. After training on genome-wide chromatin accessibility (ATAC-Seq) data across a time course of differentiation of immortalized AMSCs into mature adipocytes (before induction [D0], early differentiation [D3], advanced differentiation [D6], and terminal differentiation [D24]) (Xue et al., 2015), the Basset method identified rs56371916 as being associated with the highest difference in chromatin accessibility between the alleles (Figure 3A; Table S4), with the T allele on haplotype 1 increasing chromatin accessibility relative to the C allele on haplotype 2 in fully differentiated adipocytes (Figures 3B and S3A). The allelic difference was in the 99th percentile for all SNPs in the GWAS catalog (MacArthur et al., 2017) (empirical p = 0.0061) (Figure S3B). A third method commonly used for variant prioritization, deltaSVM (Lee et al., 2015), also highlighted the same SNP in mesenchymal cells (Table S4).

### SNP rs56371916 affects an SREBP1 binding site

**Figure 4:**
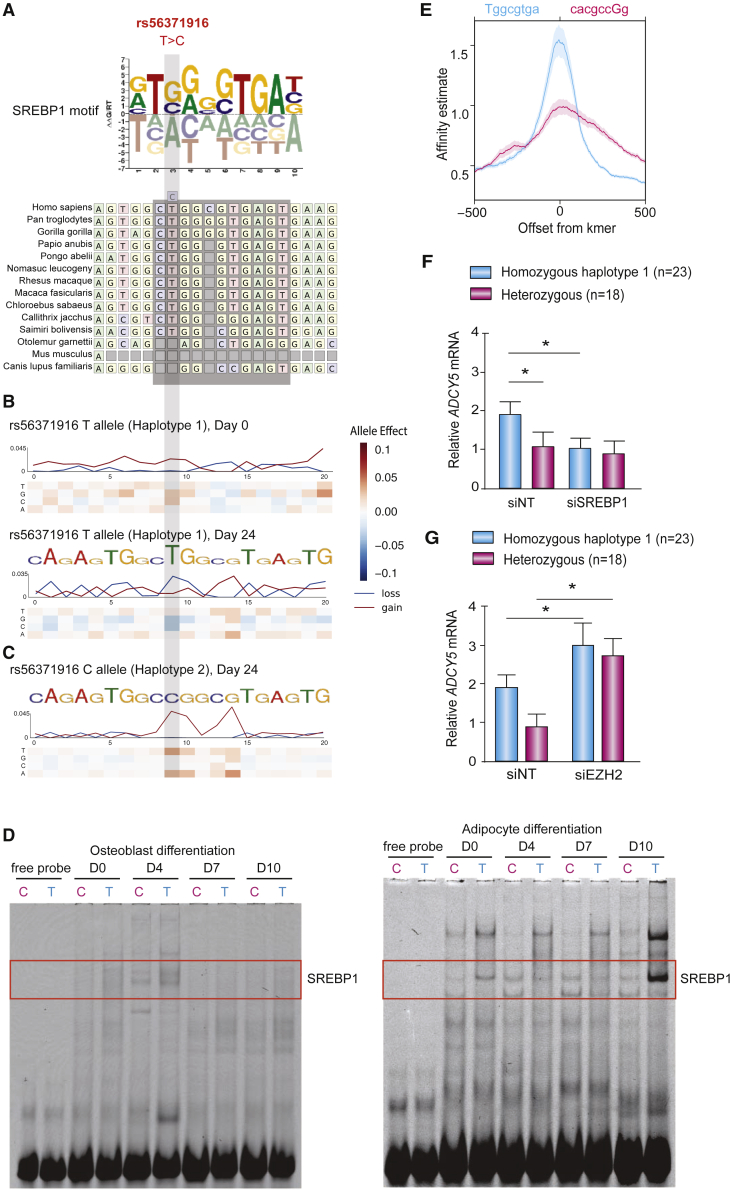
rs56371916 alters an SREBP1 binding site (A) Multi-way multiz alignment of orthologous regions of the SREBP1 motif in rs56371916 in different species. (B and C) *In silico* saturated mutagenesis for chromatin accessibility of 20 bp centered on rs56371916 for haplotype 1 (T allele, B) and haplotype 2 (C allele, C). The heatmaps display the change in predicted accessibility for any of the four possible nucleotides at day 0 and day 24 of differentiation. (D) Electrophoretic mobility shift assays (EMSAs) for 40-bp oligonucleotides centered on rs56371916 using nuclear extracts from MC3T3 osteoblast (left panel) and AMSC-derived adipocytes (right panel) at different stages of differentiation (day 0, day 4, day 7, and day 10). The EMSAs show representative blots of four independent experiments. (E) Intragenomic-replicate-method (Cowper-Sal lari et al., 2012)-based predictions of SREBP1 binding affinity. Profiles represent average SREBP1 affinities for T allele sequence of length 8 (tggcgtga, blue) and C allele sequence (cacgccgg; reverse complemented, pink) overlapping rs56371916 within a 1-kb region. (F and G) *ADCY5* gene expression in differentiating AMSC-derived osteoblasts and adipocytes treated with siRNAs targeting *SREBP1* (siSREBP1, osteoblasts and adipocytes as indicated, F), *EZH2* (siEZH2, osteoblasts, G) or non-targeting control siRNAs (siNT). Mean ± SD for n = 18 heterozygous individuals (haplotypes 1/2) and n = 23 homozygous individuals (haplotypes 1/1). Mean ± SD, ^∗^p < 0.05.

To identify the regulatory elements in the neighborhood of rs56371916, we used the Basset CNN model to analyze the effect of altering each base within a 20-bp window centered on the SNP on chromatin accessibility. We found that rs56371916 itself was predicted to have the greatest effect, with the T-to-C substitution predicted to disrupt a highly conserved second position in an SREBP motif in fully differentiated adipocytes (Mabbott et al., 2013; Weirauch et al., 2014) (Figures 4A–4C).

SREBPs are transcription factors known to play a role in cholesterol and fatty acid biosynthesis. Because SREBPs are known to play activating roles (Edwards et al., 2000), we tested whether the C allele at rs56371916 decreased enhancer activity in our luciferase assay compared with the T allele. We found that the single nucleotide T-to-C alteration in the haplotype 1 indeed decreased enhancer activity by 2.3-fold in both SGBS adipocytes (Fischer-Posovszky et al., 2007) (a frequently used *in vitro* pre-adipocyte model originated from adipose tissue from a patient with Simpson-Golabi-Behmel syndrome [SGBS]) and MC3T3 osteoblasts (Figure S3C; STAR methods). Using electrophoretic mobility shift assays (EMSAs), we also found that rs56371916 affected protein binding to the surrounding DNA sequence, with the C allele showing decreased protein binding (Figure 4D), consistent with its disruption of the predicted SREBP motif. Moreover, protein binding to the DNA sequence could be out-competed by an excess of probe containing a consensus binding sequence for SREBP (Figure S3D; Table S5). To confirm differential binding of SREBP1 to the T allele, we used the IGR method (Cowper-Sal lari et al., 2012), which compares the frequency of k-mers matching the rs56371916 T allele versus the C allele, based on publicly available SREBP1 ChIP-seq data, to estimate preferential binding affinity of SREBP1. We confirmed that SREBP1 preferentially binds to the T allele with ∼1.9-fold higher frequency (two-tailed Student’s t test p < 2.2 × 10^−6^) (Figure 4E).

Of the two mammalian sterol regulatory-element-binding proteins, SREBP1 showed high expression levels in differentiated mesenchymal cells (Figures S3E–S3G). Expression of *ADCY5* was positively correlated with expression of *SREBP1* in subcutaneous adipose tissue harboring differentiated adipocytes from 30 individuals (Cohort 2, STAR methods) (r = 0.567, p = 0.001), but not in isolated AMSCs from 24 individuals (Cohort 3, Methods), which showed a weaker inverse correlation (Figure S3E, Cohort 2, see STAR methods). *SREBP2* showed no correlation with ADCY5 expression in adipose tissue or AMSCs (Figure S3F). *ADCY5* and *SREBP1* both showed increased expression over the course of adipocyte differentiation, while *SREBP2* expression decreased (Figure S3G, yellow background). These results indicate that the relevant activating protein in adipocytes is SREBP1.

We examined the effect of siRNA-mediated knockdown of *SREBP1* on *ADCY5* gene expression in the 41 cell lines from Cohort 1, consisting of 23 haplotype 1/1 homozygotes and 18 haplotype 1/2 heterozygotes. Consistent with the notion that SREBP1 binds to the T allele more strongly than to the C allele, we found that *SREBP1* knockdown had greater effects on *ADCY5* expression in haplotype 1/1 homozygotes than haplotype 1/2 heterozygotes in both primary adipocytes (mean fold-decrease of 1.5 ± 0.1 [SEM] versus 1.2 ± 0.1, p = 3.2 × 10^−8^) and osteoblasts (1.9 ± 0.1 versus 1.1 ± 0.2, 1.8 × 10^−8^) (Figure 4F). These data indicate an activating effect of SREBP1 binding to the major T allele on haplotype 1.

In addition to studying the effect of knocking down *SREBP1* on *ADCY5* expression, we also examined the effect of depleting *EZH2*, the enzyme that catalyzes H3K27-trimethylation, in osteoblasts. We found that siRNA-mediated knockdown of *EZH2* increased *ADCY5* expression by ∼3-fold for haplotype 1/2 heterozygotes (p = 0.02) and ∼1.6-fold for haplotype 1 homozygotes (p = 0.02) (Figure 4G). This confirms that both haplotypes are under some degree of Polycomb repression, with greater repression of haplotype 2 (Figure S3H).

### SNP rs56371916 and *ADCY5* expression are associated with changes in lipid oxidation in primary human adipocytes and osteoblasts

To identify cellular processes affected by altered *ADCY5* expression in adipocytes and osteoblasts, we identified co-regulated genes in genome-wide expression data from primary human AMSCs in a cohort of 12 healthy, non-obese individuals (Cohort 3; STAR methods). The co-expressed genes were highly enriched in biological processes related to fatty acid metabolism, including fatty acid oxidation and lipolysis (Table S6), suggesting that ADCY5 might play a role in lipid oxidation processes. Positively co-regulated genes included regulators of fatty acid oxidation, such as the alcohol dehydrogenases *ADH1A* and *ADH1B*, the fatty acid transporters *CPT2* and *SLC27A2*, the acyl-CoA dehydrogenase *ACADM*, the acetyl-CoA acetyltransferase enzyme *ACAT1*, and the hydroxyacyl-CoA dehydrogenase fatty acid oxidation enzymes *HADH* and *HADHB* (Tables S7 and S8). Additional co-regulated genes encoded the rate-limiting enzyme of lipolysis *LIPE* and the lipid droplet-associated protein *PLIN1*. These correlations were not evident in isolated mature adipocytes (Table S7). We also noted several co-expressed genes relevant to bone, including *SOD1*, *KLF15*, *ZNF74*, *ZNF133*, and *ZNF485*, which are all involved in osteoblast differentiation and/or bone-related functions (Table S7). The gene with the strongest negative correlation with *ADCY5* expression levels was *LIF*, a well-known inhibitor of osteoblast differentiation (Falconi and Aubin, 2007).

We next assessed whether rs56371916, which is associated with expression levels of *ADCY5*, is also associated with expression of the putative ADCY5-regulated genes involved in lipolysis, fatty acid oxidation and osteoblast differentiation. We used qPCR to measure expression levels of key marker genes in adipocytes and osteoblasts in Cohort 1 (23 TT homozygotes and 18 CT heterozygotes). In adipocytes, we observed higher gene expression in homozygotes than heterozygotes for marker genes for lipolysis (*ATGL* [1.3-fold]*, LIPE* [2.1-fold], and *PLIN2* [1.4-fold]; Table S8). In osteoblasts, we similarly saw higher gene expression in homozygotes than heterozygotes for marker genes for fatty acid oxidation (*ACACB* [1.1-fold]*, ACAT1* [1.5-fold], and *CPT1A* [1.7-fold]) and master regulators of osteoblast differentiation (*RUNX2* [1.9-fold], *OCN* [1.4-fold], and *OSX* [1.2-fold]; Table S8). These results indicate haplotype-specific control of genes involved in lipid oxidation and osteoblast formation.

**Figure 5:**
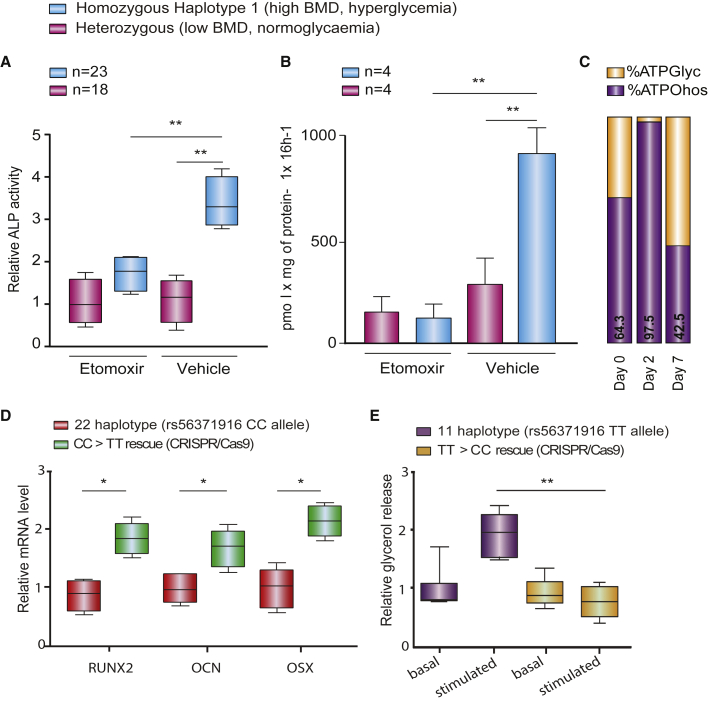
*3q21.1* risk haplotype effect on lipid oxidation in adipocytes and osteoblasts (A) Osteoblast alkaline phosphatase (ALP) activity in differentiating AMSC-derived osteoblasts treated with 100-μM etomoxir or negative control from n = 18 haplotypes 1/2 carriers and n = 23 haplotypes 1/1 carriers. Mean ± SD, ^∗∗^p < 0.01. (B) Oxidation of [14C]palmitate (0.5 mM) to 14CO_2_ in differentiating AMSC-derived osteoblasts treated with 100-μM etomoxir (carnitine palmitoyltransferase 1 antagonist) or negative control. Mean ± SD; n = 4 (haplotypes 1/2) and n = 4 (haplotypes 1/1), ^∗∗^p < 0.01. (C) Seahorse XF24 Flux analysis of murine bone marrow cells undergoing osteoblast differentiation at days 0, 2, and 7 of differentiation. Bars display relative contribution of glycolysis and oxidative phosphorylation to ATP generation in the cells. (D) Quantification of osteoblast differentiation marker gene expression from qPCR analysis in isogenic AMSC-derived osteoblasts with genotype TT and genotype CC at rs56371916. Mean ± SD, n = 3. ^∗^p < 0.05. (E) Quantification of basal and catecholamine-stimulated lipolysis rate in isogenic AMSC-derived adipocytes with genotype TT and genotype CC at rs56371916. Mean ± SD, n = 3, ^∗^p < 0.05, ^∗∗^p < 0.01.

These differences in gene expression were associated with cellular signatures relevant to hyperglycemia and bone density. In adipocytes, adrenergic lipolysis rate and fatty acid release as measured by catecholamine-stimulated glycerol release were 1.9-fold higher in haplotype 1 homozygotes than heterozygotes (p = 0.0012, Figure S3I). Increased release of fatty acids from fat tissue is a hallmark of hyperglycemia (Guilherme et al., 2008; Shulman, 2014; Girousse et al., 2013). In osteoblasts, we found greater osteoblast differentiation in haplotype 1 homozygotes than heterozygotes (3-fold change, p = 0.0014, Figure 5A, Cohort 1) using ALP activity, a surrogate of increased bone formation (Farley and Baylink, 1986). Furthermore, we observed a greater extent of fatty acid oxidation in osteoblasts from 4 haplotype 1 homozygotes compared with 4 heterozygotes using radiolabeled palmitic acid oxidation assays (3-fold change, p = 0.003, Figure 5B, Cohort 4, STAR methods). This effect on fatty acid oxidation was greater specifically during early stages of osteoblast differentiation (day 3 of osteoblast differentiation, Figure S3J).

To better understand the role of lipid oxidation in osteoblasts, we performed osteoblast differentiation assays in AMSCs from Cohort 1, treating the cells with etomoxir, which inhibits the rate-limiting enzyme in fatty acid oxidation carnitine palmitoyltransferase 1 (CPT-1) during early differentiation. The inhibitor revealed a dramatically lower extent of osteoblast differentiation for the haplotype 1 homozygotes compared with haplotype 1/2 heterozygotes (p = 0.005, Figure 5A), while no effect was observed in heterozygotes.

These data suggest that greater osteoblast differentiation depends on fatty acid oxidation at some stage in differentiation. While fatty acid utilization has previously been identified as an important process to support osteoblastogenesis, we sought to further test whether the metabolic phenotype changed throughout the differentiation profile. We found that ATP was primarily being generated via oxidative phosphorylation at day 0 (64.3%) and day 2 (97.5%) of differentiation, which are time points consistent with early osteo-progenitor cells (Figure 5C). Interestingly, later in osteoblast differentiation, the more mature osteoblasts (day 7) demonstrated a more glycolytic phenotype (Figure 5C). The switch was accompanied by increased expression by day 2 of genes encoding fatty acid transporters, including *CD36, SLC27A1 (aka FATP1)*, and *SLC27A3 (aka FATP3)* and cytoplasmic lipases, including *ATGL* (*aka PNPLA2*)*, LIPE (aka HSL)*, and *MGLL* (Figure S3K). Taken together these data indicate that during the early stages of osteoblast differentiation, osteo-progenitor cells depend on the use of fatty acids as an energy source; however, their metabolic profile switches toward glycolysis in the more mature osteoblast.

### Changing *ADCY5* expression affects lipid oxidation in primary adipocytes and osteoblasts

To show directly that ADCY5 levels regulate lipolysis in adipocytes and lipid oxidation-dependent differentiation processes in osteoblasts, we altered *ADCY5* expression in those cells. In adipocytes, we found that using lentiviral overexpression to increase *ADCY5* levels by 60% led to elevated rates of catecholamine-stimulated lipolysis and fatty acid release, as measured by glycerol release in 4 heterozygotes compared with 4 homozygotes for haplotype 1 (1.9-fold, SD = 0.8, Figure S3L, Cohort 4). In osteoblasts, we found that using pooled siRNAs to decrease *ADCY5* expression by 56% led to a major decrease in osteoblast differentiation as assessed by marker genes. Larger effects were seen in the 4 homozygotes for haplotype 1 compared with the 4 heterozygotes (*RUNX2* 2.9- versus 1.3-fold*, OCN* 3.2- versus 1.3-fold, and *OSX* 3.7- versus 1.5-fold; SEM = 0.1), consistent with the notion that high levels of *ADCY5* expression increase osteoblast differentiation in haplotype 1 carriers (Figure S3M).

### Genome editing of rs56371916 confirms that this variant affects *ADCY5* expression, lipid oxidation, and osteoblast differentiation

To confirm directly that the haplotype-specific effects on *ADCY5* gene expression and cellular properties described above are mediated by rs56371916, we performed CRISPR-based genome editing to make isogenic changes at this SNP. We edited AMSCs from a homozygote for haplotype 2 (genotype CC at rs56371916) to create isogenic AMSCs with genotype TT. Following osteoblast induction, TT homozygous cells showed higher *ADCY5* expression levels (1.6-fold) (Table S9) and higher expression of osteoblast differentiation marker genes (*RUNX2*, osteocalcin [*OCN*], and osterix [*OSX*]; 1.5-, 1.8-, and 2.1-fold, respectively; Figure 5D).

We also edited AMSCs from a homozygote for haplotype 1 (genotype TT at rs56371916) to create isogenic AMSCs with genotype CC. Following adipocyte induction, CC homozygous cells showed reduced expression of *ADCY5* (1.4-fold) and lipolysis marker genes (1.5- to 1.9-fold), as measured by qPCR (Table S9), as well as a reduced rate of catecholamine-stimulated lipolysis compared with CC homoozygotes (2.1-fold, Figure 5E). Our genome editing results in primary adipocytes and osteoblasts prove that rs56371916 has a direct effect on *ADCY5* gene expression and cellular phenotypes relevant to FNBMD and glucose homeostasis. While we cannot rule out, however, that rs56371916 may also affect other additional cell types or that additional variants at the *3q21.1* locus may also have effects on bone and glycemic traits, we show in this study that the rs56371916 T allele causally affects cellular phenotypes in adipocytes and osteoblasts that are relevant to the studied traits, i.e., hyperglycemia and FNBMD.

## Discussion

While GWASs have largely focused on individual phenotypes, there is growing evidence that many loci have pleiotropic effects, being associated with multiple traits (Bulik-Sullivan et al., 2015; Pickrell et al., 2016). Studying pleiotropic effects of loci across cell types and tissues is thus important—and may also be useful for discovering the causal variants and their mechanism of action. In this study, we focused on shared genetics between BMD and glycemic traits, to help explain the molecular underpinnings of a clinically recognized link between T2D and bone metabolism (Leslie et al., 2012). Briefly, using GWAS summary statistics for BMD and glycemic traits, we found a pleiotropic locus at *3q21.1*, associated with FNBMD and fasting glucose levels. We showed that the variant rs56371916, a strongly associated SNP in an intronic region of *ADCY5*, plays a causal role in processes related to these phenotypes by affecting the binding affinity of SREBP1, shifting the balance between poised and repressed chromatin in mesenchymal cells. We further showed that the genotype rs56371916 affects *ADCY5* expression in both adipocytes and osteoblasts, which results in altered lipid metabolism. We validated our results by directly manipulating the upstream regulator *SREBP1* and the target gene *ADCY5* by siRNA-mediated knockdown and overexpression experiments and by performing CRISPR-mediated genome editing on rs56371916 in primary human adipocytes and osteoblasts. We note that the rs56371916 T allele, which associates with increased BMD and hyperglycemia, showed a trend for association with decreased fracture risk in a recent GWAS study (rs56371916 C/T, OR: 0.98, p = 0.08) (Morris et al., 2019) and was shown to be associated with an increased risk of T2D (rs56371916 C/T, OR: 1.08, p = 3.6 × 10^−27^).

ADCY5 is a member of the membrane-bound adenylate cyclase family of enzymes that mediates G-protein-coupled receptor signaling through the synthesis of the metabolic messenger cAMP (Defer et al., 2000). Among other roles, cAMP plays a key role in lipolysis in adipocytes during fasting and stress, controlling the release of free fatty acids into the bloodstream. *ADCY5* is among several adenylate cyclases expressed at high levels in mesenchymal cells (Mabbott et al., 2013). Coherent with a pleiotropic role of *ADCY5*, variants in *ADCY5* have further been associated with 2-h glucose challenge (Saxena et al., 2010), fasting glucose, T2D (Dupuis et al., 2010; Fuchsberger et al., 2016), and with beta cell insulin secretion (Dayeh et al., 2013). In light of pleiotropy, this locus is particularly interesting as previous work has convincingly shown that the lead SNP of the univariate T2D GWAS signal, rs11708067, which is also the variant with the highest posterior probability at that locus (Thurner et al., 2018), associates with an altered chromatin state (Roman et al., 2017; Thurner et al., 2018), DNA methylation (Olsson et al., 2014), and transcript expression of *ADCY5* in pancreatic cells (van de Bunt et al., 2015; Dayeh et al., 2013; Roman et al., 2017; Thurner et al., 2018; Hodson et al., 2014) and resides in an islet enhancer which interacts with the *ADCY5* promoter (Thurner et al., 2018). Together, these data show that even for a well-characterized locus as the *ADCY5* locus, which would provide sufficient evidence to mechanistically explain the T2D association in pancreatic islets, it is meaningful to test other variant(s) that might exert their pleiotropic effects in multiple tissues as we show here for adipose and bone. This concept is further reinforced by a recent study reporting that around 90% of loci contain associations with multiple traits across multiple trait domains (Watanabe et al., 2019).

Our work implicates adenylate cyclase 5 in bone- and glycemia-related phenotypes. We examined our list of other bivariate loci for these phenotypes in the GEFOS and MAGIC consortium data, as well as loci with pleiotropic effects on bone- and adipose-related traits in the UK Biobank database (http://big.stats.ox.ac.uk). Intriguingly, we noted that the low-frequency missense variant, rs3730071, in another adenylate cyclase, *ADCY6*, showed genome-wide significant effects on both BMD (p = 2.2 × 10^−19^) and fat mass (p = 1.6 × 10^−09^) (http://big.stats.ox.ac.uk/variant/12-49168798-C-A). This observation provides additional support for the role of adenylate cyclases on pleiotropy of bone and adipose.

Our study sheds light on a critical role of ADCY5 in fatty acid oxidation in adipocytes and osteoblasts. The locus has previously been associated with high-density lipoprotein cholesterol (HDL) and total cholesterol (TC) plasma concentrations in a multi-ethnic GWAS (Hoffmann et al., 2018), further implicating a role of lipid metabolism at the ADCY5 locus. While the physiological impact of lipid oxidation in adipocytes has been investigated in earlier studies, little research has focused on the role of fatty acid oxidation in osteoblasts and how this might impact osteoblast differentiation. Acquisition of peak BMD is dependent on extensive osteoblast progenitor differentiation and is metabolically demanding. Our work shows for the first time that osteo-progenitor cells preferentially metabolize fatty acids and that inhibition of fatty acid oxidation during early stages of differentiation is sufficient to stall osteoblast differentiation programs. This context-specific feature of osteoblast bioenergetics supports the notion that adenylate cyclase activity, which is essential for lipolysis, is central in osteoblast differentiation and ultimately BMD regulation. Consistent with our findings, partial loss-of-function of the GNAS complex (G protein alpha subunit), which directly stimulates adenylate cyclases, results in low bone mass and a lack of adipose tissue (Balasubramanian et al., 2015).

Our results from genetic association in human populations and experimental studies of adipocytes and osteoblasts *in vitro* provide strong evidence that expression levels of *ADCY5* affect T2D and BMD, offering a possible explanation for increased BMD in individuals with T2D. Extending our findings beyond osteoblast differentiation may also shed light on why the higher density bone in T2D is associated with greater fragility and may have implications for developing treatment regimens for either trait without adverse effects on the other. Future studies, however, will be needed to carefully study organismal physiology in both humans and genetically engineered animal models. We further note that the *3p21.1* risk locus studied in this work is one of multiple genetic risk loci which appear to associate with both increased risk for T2D and increased BMD. Lastly, in this work we have limited our bivariate genetic association analyses on BMD related traits and glycemic traits. In light of recent epidemiological evidence linking obesity to increased BMD and fragility (reviewed in Benova and Tencerova, 2020), it will be of particular interest to expand on our analyses and model the genetic architecture of obesity, T2D, and BMD more broadly using multivariate large-scale genetic association studies.

### Limitations of study

One limitation of our study is a limited set of CRISPR-edited AMSCs for rs56371916 TT and CC allele carriers. This is largely due to technical difficulties and limitations of editing single nucleotides in primary pre-adipocytes with current genome editing technologies. Specifically, AMSCs have very low homology directed repair (HDR) rates and are not easily amenable to clonal expansion. In this study, we used CRISPR-Cas9 to edit single nucleotides in 2 individuals (one homozygous risk and one homozygous non-risk allele carrier) with coherent results across the two individuals on target gene expression, lipolysis, and osteoblast differentiation. Another limitation of the GWAS data that generated the bivariate signal is that data came from individuals from European ancestry. Findings might not replicate in other ancestries. Lastly, our finding is discovering a locus that conveys increased femur neck and lumbar spine BMD as well as T2D, which is completely compatible with the observation that individuals with T2D have preserved or even increased BMD as measured by dual-energy X-ray absorptiometry (DEXA) scans. Despite the increased bone density, an increase in bone fragility had been observed, as well as an increased risk for certain fragility fractures. Future studies of the *3q21* locus with high resolution peripheral quantitative computed tomography imaging would provide additional insights into skeletal mechanisms of the rs56371916 functional variant.

## STAR★methods

### Key resources table

**Table:** 

REAGENT or RESOURCE	SOURCE	IDENTIFIER
**Antibodies**		

H3K27me3 polyclonal antibody	Diagenode	C15410069
Anti-Histone H3 (acetyl K27) antibody - ChIP Grade	Abcam	ab4729

**Biological samples**		

Mouse C57BL/6J primary bone marrow stromal cells (BMSCs)	Cliff Rosen lab, Maine Medical Center	N/A
primary AMSC	TUM	N/A
Human adipose tissue	University Bergen	N/A

**Chemicals, peptides, and recombinant proteins**		

TRIzol	Invitrogen	15596026
Oil Red O solution	Merck	O1391
SYBR Green	Invitrogen	S7563
Biotin	Merck	B4639
pantothenic acid	Merck	P5155
Transferrin human	Merck	T3309
hEGF	Sigma-Aldrich	E9644
hFGF	Sigma-Aldrich	F0291
Hydrocortisone	Sigma-Aldrich	H0888
Transferrin	Sigma-Aldrich	T8158
Triiodo-L-thyronine	Sigma-Aldrich	T6397
Rosiglitazone	Sigma-Aldrich	R2408
Dexamethasone	Sigma-Aldrich	D4902
3-Isobutyl-1-methylxanthine	Sigma-Aldrich	I5879
β-Glycerophosphate	Sigma-Aldrich	G9422
Lipofectamine 2000	Invitrogen	11668019
Indomethacin	StemCell	73942
D-Luciferine K salt (potassium salt)	PJK GmbH	N/A
Coelenterazine	PJK GmbH	N/A
Isoproterenol	Sigma-Aldrich	I6504
Palmitic acid, [1-14C]-	PerkinElmer	NEC075H050UC
SIGMAFAST BCIP/NBT	Sigma-Aldrich	B5655
Alizarin-Red Staining Solution	Sigma-Aldrich	TMS-008
Oleic acid	Sigma-Aldrich	O1008
UK-5099	Sigma-Aldrich	PZ0160
BPTES	Sigma-Aldrich	SML0601
Etomoxir	Sigma-Aldrich	E1905
Sodium butyrate	Sigma-Aldrich	B5887

**Critical commercial assays**		

Tet-On Advanced Inducible Gene Expression System	BD Biosciences	
DNeasy Blood & Tissue Kit	QIAGEN	69506
RNeasy Lipid Tissue Mini Kit	QIAGEN	74804
High-Capacity cDNA Reverse Transcription Kit	Applied Biosystems	4368814
MACSelect Transfected Cell Selection kit	Miltenyi	130-091-988
Q5 Site-Directed Mutagenesis Kit	New England Biolabs	E0554S
Glycerol-3-Phosphate Assay Kit	Sigma-Aldrich	MAK207

**Deposited data**		

FNBMD GWAS summary statistics	GEFOS Consortium	
GWAS glycaemic trait sample	MAGIC GWAS Consortium	
Hi-C data from human H1-hESC derived mesenchymal stem cell cultured cells	Dixon et al., 2015	SRR1030739-SRR1030744

**Experimental models: cell lines**		

Human Huh7 hepatoma	Hans Hauner lab, TUM	N/A
mouse C2C12 myoblasts	ATCC	N/A
HT22 neuronal cells	Millipore Sigma	N/A
Normal Human Articular Chondrocytes (NHAC-kn)	Clonetics	N/A
human K562 lymphoblastoid	Hans Hauner lab, TUM	N/A
human pre-adipocyte SGBS (Simpson–Golabi–Behmel Syndrome) cell line	Martin Wabitsch, University of Ulm	N/A
MC3T3 osteoblasts	ATCC	N/A

**Oligonucleotides**		

Genotyping primers	Eurofins genomics	N/A
EMSA probes	Eurofins genomics	N/A
ATAC-seq barcodes	IDT	N/A

**Recombinant DNA**		

hCas9 plasmid	Addgene	41815
gRNA cloning vector	Addgene	41824
pGL4.22 firefly luciferase reporter vector	Promega	E6771
pRL-Ubi	Promega	E2241
TK promoter control vector	Hans Hauner lab, TUM	N/A
doxycycline-inducible Tet-On Advanced Inducible Gene Expression System	Takara	631069
pMACS plasmid 4.1	Milteny Biotech	130-091-886

**Software and algorithms**		

CP-ASSOC	http://hal.case.edu/∼xxz10/zhu-web/	N/A
MTAG	https://github.com/JonJala/mtag	N/A
eLX package	https://www.npmjs.com/package/elx	N/A
WashU Epigenome Gateway	interactive session 2apaIcl6nH	N/A
bowtie2	http://bowtie-bio.sourceforge.net/bowtie2/index.shtml	N/A

**Other**		

DMEM	Gibco	10569010
Penicillin-Streptomycin (10.000 U/ml)	Gibco	15140122
Fetal Bovine Serum	Gibco	26140079
MCDB131 Medium	Gibco	10372019
DMEM/F-12	Gibco	11320033
MEM α	Gibco	12571063
Passive Lysis 5X Buffer	Promega	E1941
HumanRef-8 v.3 and Ref-12 v.4 BeadChip microarrays	Illumina	
Tagmentation DNA (TD) Buffer	Nextera	FC-121-1031
Protease Inhibitor Cocktail	Sigma	P8340
MNase-based Enzymatic Shearing Cocktail	Active Motif	103295

### Resource availability

#### Lead contact

Further information and requests for resources and reagents should be directed to and will be fulfilled by the Lead Contact, Melina Claussnitzer (melina@broadinstitute.org).

#### Materials availability

All stable reagents generated in this study -- except for those that we require to maintain the stock -- will be made available on request, but we may require a payment and/or a completed Materials Transfer Agreement if there is potential for commercial application.

#### Data and code availability

The datasets generated during this study are available at dbGAP, PRJNA664585

### Experimental model and subject details

#### Human samples

##### Subjects and primary tissues and cell culture

Clinical characteristics of subjects from which adipose tissue, isolated floating adipocytes and AMSCs were derived are outlined in the Table below. Human adipose tissue was obtained with informed, written consent from each subject, and approval by the local ethics committee of the Faculty of Medicine of the Technical University of Munich, Germany, or the Regional Committee for Medical Research Ethics (REK) of Haukeland University Hospital, Bergen, Norway. We used three different samples, including heterogeneous whole adipose tissue, adipose-derived mesenchymal stem cells (AMSCs) and isolated floating adipocytes. Primary human AMSC cell cultures were obtained from subcutaneous adipose tissue of healthy European subjects 20 to 50 years of age and with a normal body-mass index (BMI) (20 to 24.9 kg/m^2^). Cells were isolated as previously described (Claussnitzer et al., 2014; van Harmelen et al., 2005) with some modifications (see below). Genotyping was done by MassARRAY (Sequenom), Omni express (Illumina) or Sanger Sequencing. In addition, for direct RNA isolation and gene expression analysis, we obtained whole adipose tissue and AMSCs from non-genotyped healthy non-obese subjects undergoing elective surgeries (BMI 18 to 28 kg/m^2^) or severely obese European subjects undergoing bariatric surgery (BMI 35 to 52 kg/m^2^) (25 to 67 years of age), as described previously (Veum et al., 2012). The subjects were genotyped for both the identified GWAS index SNP (rs2124500) and the identified causal variant rs56371916.

Clinical characteristics of subjects with available tissue samples included in the study.

**Table:** 

Cohort	Genotype	Sex (Male/Female)	Age (Years)	BMI (kg/m2)	Available Sample Types	Figure/Table
Cohort 1	Haplotype 1/1	2/21 (n= 23)	35.4 ± 10.4	23.5 ± 1.8	AMSCs	Figures 2E–2G, 4F, 4G, 5A, S4A, S3I, and S3M; Tables S3 and S8
	Haplotype ½	2/16 (n = 18)	38.6 ± 11.2	22.9 ± 1.7		
Cohort 2	ND	11/19 (n = 30)	43.1 ± 14.1	39.8 ± 15.5	Whole SC/OM adipose tissue	Figures S3E and S3F
Cohort 3	ND	9/15 (n = 24)	43.3 ± 11.0	33.3 ± 11.4	Mature adipocytes, AMSCs	Figures S3E and S3F; Tables S6 and S7
Cohort 4 (Sub-Cohort of cohort 1)	Haplotype 1/1	0/4 (n = 4)	35.8 ± 8.1	22.9 ± 1.0	AMSCs	Figures 5B, S3J, and S3L
	Haplotype ½	0/4 (n = 4)	31.8 ± 4.9	23.4 ± 1.4		
CRISPR Genome Editing	Haplotype 1/1 (TT at rs56371916)	0/1 (n = 1)	28	20.8	AMSCs	Figure 5D; Table S9
	Haplotype 2/2 (CC at rs56371916)	0/1 (n = 1)	31	22.1	AMSCs	Figure 5E; Table S9

BMI, body-mass index (kg/m^2^); AMSC, adipose mesenchymal stem cells; OM, omental adipose tissue; SC, subcutaneous adipose tissue.

### Method details

#### Genotyping

Genomic DNA was isolated from blood using the DNeasy Blood&Tissue Kit from Qiagen according to the manufacturer’s protocol. A 250bp fragment surrounding rs56371916 and rs2124500 was generated by PCR using the primers indicated below. The PCR product was sequenced using the Sanger sequencing services from Genewiz using the indicated primers.

**Table:** PCR amplification primers

rs56371916_for	CTGAGTGGAAATCACCGCCA
rs56371916_rev	GTGAAAAGTAATCTTCCTGCCTGG
rs2124500_for	GTAGTGGCACTGGAACTTGA
rs2124500_rev	GTGGGTCAGTCCCAAATCTT

**Table:** Sequencing primers

rs56371916_seq_for	AGTGGAAATCACCGCCAG
rs2124500_seq_for	AGTGGCACTGGAACTTGAAC

#### Culture and differentiation of primary human AMSCs

Human liposuction material used for isolation of AMSCs was obtained from a collaborating private plastic surgery clinic Medaesthetic Privatklinik Hoffmann & Hoffmann in Munich, Germany. Harvested subcutaneous liposuction material was filled into sterile 1-L laboratory bottles and immediately transported to the laboratory in a secure transportation box. The fat was aliquoted into sterile straight-sided wide-mouth jars, excluding the transfer of liposuction fluid. The fat was stored in cold Adipocyte Basal medium (AC-BM) at a 1:1 ratio of fat to medium at 4°C to be processed the following day. Additionally, small quantities of the original liposuction material would be aliquoted into T-25 flasks at a 1:1 ratio of fat to medium as controls to check for contamination. These control flasks were stored in the 37°C incubator and were not processed. Krebs-Ringer Phosphate (KRP) buffer was prepared containing 200 U/ml of collagenase and 4 % heat shock fraction BSA and sterilized by filtration using a BottleTop Filter 0.22μM. When the adipose reached room temperature, 12.5 ml of liposuction material was aliquoted into sterile 50-ml tubes with plug seal caps. The tubes were filled to 47.5 ml with warm KRP-BSA-collagenase buffer and the caps were securely tightened and wrapped in Parafilm to avoid leakage. The tubes were incubated in a shaking water bath for 30 minutes at 37°C with strong shaking. After 30 minutes, the oil on top was discarded and the supernatant was initially filtered through a 2000-μm nylon mesh. The supernatant of all tubes was combined after filtration and centrifuged at 200xg for 10 minutes. The supernatant was discarded and each pellet was resuspended with 3ml of erythrocyte lysis buffer, then all the pellets were pooled and incubated for 10 minutes at RT. The cell suspension was filtered through a 250-μm nylon mesh and then through 150-μm nylon mesh, followed by centrifugation at 200 × g for 10 minutes. The supernatant was discarded and the pellet containing pre-adipocytes was resuspended in basal growth media (DMEM/F12 with 1% penicillin/streptomycin (P/S) 33-μM biotin, 17μM Pantothenic Acid, and 10% FCS), seeded into T75 cell culture flasks and placed in an incubator (37°C, 5% CO_2_). The next day, medium was changed to expansion medium (DMEM/F12 supplemented with 2.5% FCS, 1% penicillin/streptomycin, 33μM biotin, 17μM Pantothenic Acid, 132nM insulin, 10ng/ml EGF, and 1ng/ml FGF) and cells grown until they reached near confluence. Cells were passaged twice before aliquots of 500-K cells were viably frozen. For adipogenic differentiation, AMSCs were seeded into 6- or 12-well plates and grown until they reach confluence. At confluence, AMSCs were induced to differentiate by supplementing DMEM/F12 with 1% penicillin/streptomycin (P/S), 33-μM biotin, 17μM Pantothenic Acid, 66nM insulin, 100nM cortisol, 10μg/ml transferrin, 1nM triiodo-L-thyronin (T3), 2μM rosiglitazone, 25nM dexamethasone and 0.5mM IBMX. Osteogenic differentiation was induced by aMEM with 10%FCS, 1% P/S, 10mM b-glycerophosphate and 400nM hydrocortisone.

#### Culture and differentiation of immortalized AMSCs

Immortalized AMSCs (iAMSCs) were received from Prof Yu-Hua Tseng (Harvard Medical School, Joslin Diabetes Center). The cells were previously isolated and immortalized from human subcutaneous white adipose tissue of a female subject, aged 56 with a BMI of 30.8. Culture and differentiation were performed following the protocol from the originating lab as described in (Xue et al., 2015). Briefly, pre-adipocytes were cultured in DMEM GlutaMAX (Gibco, 10569010) supplemented with 10% fetal bovine serum (Gibco 10082–147) and 1% penicillin/streptomycin (P/S) (5,000 U/mL) (Gibco, 15070063) at 37°C and 5% CO_2_. For differentiation, cells were treated with 0.25% trypsin (Gibco), counted using an automatic cell counter and 100-K cells per well were seeded in a 12-well plate. Once cells reached confluency, differentiation was induced by adding freshly prepared adipogenic induction medium to cells (DMDM/High glucose supplemented with 10% FBS, 1% P/S, 33-μM biotin, 0.5 μM Human insulin, 17 μM Pantothenate, 0.1 μM dexamethasone, 2 nM 3,3′,5-triiodo-L-thyronine (T3), 500 μM Isobutyl methylxanthine (IBMX), and 30 μM indomethacin). Induction media were replaced every three days for 24 days, until fully differentiated.

#### RNA preparation and qPCR

Total RNA was extracted with Trizol (Invitrogen) or RNeasy Lipid Tissue Kit (Qiagen). cDNA was synthesized with High-Capacity cDNA Reverse Transcription Kit (Applied Biosystems). qPCR was performed using SYBR Green with 60°C annealing temperature. Relative gene expression was calculated by the delta delta Ct method. Target gene expression was normalized to expression of *HPRT* (human) (de Kok et al., 2005)

**Table:** PCR amplification primers

Gene Symbol	Gene Name	Primer Sequence (5′–3′)	
Mouse			
CD36	Cluster of differetiation 36	Fwd:	GGA ACT GTG GGC TCA TTG C
		Rev:	CAT GAG AAT GCC TCC AAA CAC
Fatp1	Fatty acid transport protein 1; or solute carrier family 27 member 1 (Slc27a1)	Fwd:	CCG TAT CCT CAC GCA TGT GT
		Rev:	CTC CAT CGT GTC CTC AAT GAC
Fatp3	Fatty acid transport protein 3; or solute carrier family 27 member 3 (Slc27a3)	Fwd:	AGG GTG ACA GTG TTC CAG TAC ATT
		Rev:	TGG TCA CAC TCT GCC TTG CT
Hprt1	Hypoxanthine phosphoribosyltransferase 1	Fwd:	GCC TAA GAT GAG CGC AAG TTG
		Rev:	TAC TAG GCA GAT GGC CAC AGG
Lipc	Lipase C; or Hepatic lipase	Fwd:	GAC GGG AAG AAC AAG ATT GGA
		Rev:	GGA CGT TCC CTC AAA CAT AGG
Lipe	Lipase E; or Hormone sensitive lipase	Fwd:	CAT CAA CCA CTG TGA GGG TAAA G
		Rev:	AAG GGA GGT GAG ATG GTA ACT
Lpl	Lipoproetin lipase	Fwd:	AGC AGG AAG TCT GAC CAA TAA
		Rev:	ATC AGC GTC ATC AGG AGA AAG
Mgll	Monoglyceride lipase	Fwd:	TTC TGC TGA CCG GCT TTG
		Rev:	GAC GTG ATA GGC ACC TTC ATA C
Pnpla2	Patatin like phospholipase domain containing 2; or adipose triglyceride lipase; or desnutrin	Fwd:	GAC GGA GAG AAC GTC ATC ATA TC
		Rev:	CCA CAG TAC ACC GGG ATA AAT
Human			
HPRT	Hypoxanthine phosphoribosyltransferase 1	Fwd:	CATTATGCTGAGGATTTGGAAAGG
		Rev:	CTTGAGCACACAGAGGGCTACA
RUNX2	Runt-related transcription factor 2	Fwd:	GCCTTCAAGGTGGTAGCCC
		Rev:	AAGGTGAAACTCTTGCCTCGTC
OCN	Osteocalcin	Fwd:	AGCAAAGGTGCAGCCTTTGT
		Rev:	GCGCCTGGGTCTCTTCACT
OSX	Osterix	Fwd:	CCCCACCTCTTGCAACCA
		Rev:	CCTTCTAGCTGCCCACTATTTCC
CEBPA	CCAAT enhancer-binding protein alpha	Fwd:	GGAGCTGAGATCCCGACA
		Rev:	TTCTAAGGACAGGCGTGGAG
PPARG	Peroxisome proliferator-activated receptor gamma	Fwd:	GAAAGCGATTCCTTCACTGAT
		Rev:	TCAAAGGAGTGGGAGTGGTC
ADIPOQ	Adiponectin	Fwd:	GGTGAGAAGGGTGAGAAAGGA
		Rev:	TTTCACCGATGTCTCCCTTAG
ACC	Acetyl Coenzyme A Carboxylase B	Fwd:	GCCATTGGTATTGGGGCTTAC
		Rev:	CCCGACCAAGGACTTTGTTG
ACAT1	Acetyl-CoA Acetyltransferase 1	Fwd:	GATGAAGGAAGGCTGGTGC
		Rev:	GGAAGCTGGTGGCAGTGTAT
CPT1	Carnitine Palmitoyltransferase 1	Fwd:	CATGTATCGCCGCAAACTGG
		Rev:	CCTGGGATGCGTGTAGTGTT
ATGL	Adipose triglyceride lipase	Fwd:	CTGCCGGGAGAAGATCAC
		Rev:	AGAGGGTGGTCAGCAGGTC
LIPE	Lipase E	Fwd:	GCGGTGGCGAAAAGACAAG
		Rev:	GGTCCAGGTCAAAGAGGTG
PLIN2	Perilipin 2	Fwd:	TGAGATGGCAGAGAACGGTGTGAA
		Rev:	TTGCGGCTCTAGCTTCTGGATGAT

#### Oil Red-O staining

Lipid droplets are lipid-storage organelles predominantly present in differentiated adipocytes. ORO selectively stains neutral lipids, such as cholesteryl esters, triglycerides, and fatty acids, in cultured differentiated adipocytes, serving as a good measurement for the degree of differentiation. At day 10 of differentiation, the culture medium was removed and cells were carefully washed with PBS. The cellular monolayer was then covered with 3.7% formaldehyde to fix the cells. After 1 hr, the formaldehyde was removed and cells were stained with ORO staining solution (0.3% Oil-Red-O in 60ml Isopropanol and 40 ml H2O, filtered before use) and left to incubate for 1 hr. Afterwards, the ORO solution was removed and cells were washed twice and kept in PBS. Differentiated adipocytes full with lipid droplets will show a strong red color.

#### Luciferase expression constructs

For the promoter construct, we cloned a 752 bp thymidine kinase (TK) promoter upstream of the firefly luciferase gene into the EcoRV and BglII sites of the pGL4.22 firefly luciferase reporter vector (Promega). We then subcloned the following non-coding genomic regions upstream of the TK promoter into the KpnI and SacI sites of the pGL4.22-TK vector in forward orientations: (i) To assay haplotype enhancer activity we used 10 kb genomic regions flanking rs56371916 synthesized as plasmid vectors (Life Technologies) for both the ancestral and derived haplotype using HapMap individuals information; (ii) To assay rs56371916 allelic activity 1 kb genomic regions flanking rs56371916 carrying both alternate alleles at rs56371916 were synthesized. We cloned genomic DNA segments upstream of the TK promoter into the KpnI and SacI sites of the pGL4.22-TK vector in forward orientation. All constructs were verified by Sanger sequencing of plasmids. We performed transfection in different cell types as described below.

#### Transfection in cell cultures

Human Huh7 hepatoma, mouse C2C12 myoblasts, HT22 neuronal cells, Clonetics™ Normal Human Articular Chondrocytes (NHAC-kn), and human K562 lymphoblastoid lymphoblastoid cell lines were cultured in DMEM medium (supplemented with P/S and 10 % FBS). The human pre-adipocyte SGBS (Simpson–Golabi–Behmel Syndrome) cell line was cultured as previously described (Claussnitzer et al., 2014) in DMEM/F12 (1:1) medium (supplemented with 10% FCS, 17 μM biotin, 33 μM pantothenic acid and 1% P/S). To promote adipose differentiation of the SGBS cell line, cells were grown to confluence. For induction of adipocyte differentiation cells were cultured in serum free MCDB-131/DMEM/F12 (1:2) medium supplemented with 11 μM biotin, 22 μM pantothenic acid, 1% P/SP/S, 10 μg/ml human transferrin, 66 nM insulin, 100 nM cortisol, 1nM triiodothyronine, 20 nM dexamethasone, 500 μM 3-isobutyl-1-methyl-xanthine (Serva, Germany) and 2 μM rosiglitazone (Alexis, Germany). All cells were maintained at 37°C and 5% CO_2_. Huh7 cells (96-well plate, 1.1 x 10^4^ / well) were transfected one day after plating with approximately 90% confluence, K562 cells (12-well plate, 8 x 10^4^ / well) were transfected three days after plating with approximately 90% confluence, SGBS adipocytes (12-well plate, 8 x 10^4^ / well) were transfected at day eight after the induction of differentiation with approximately 80% confluence and C2C12 cells (12-well plate, 2 x 10^5^ / well) were transfected at day four after induction of differentiation with approximately 90% confluence. MC3T3 osteoblasts were seeded (seeding density: 250,000 cells/well) in a 6-well plate. Cells were differentiated using αMEMmedium supplemented with 10% FBS, 1% P/S, 50 ug/ml ascorbic acid, and 10 mM beta-glycerophosphate. Huh7 were transfected with 0.5 μg of the respective firefly luciferase reporter vector and 1 μl Lipofectamine 2000 transfection reagent (Invitrogen, Darmstadt, Darmstadt, Germany), differentiated C2C12 myocytes were transfected with 1 μg of the respective pGL4.22-TK construct and 2 μl Lipofectamine reagent, and both K562-cells and differentiated SGBS adipocytes were transfected with 2 μg of the respective pGL4.22-TK construct and 2 μl Lipofectamine reagent. The firefly luciferase constructs were co-transfected with the ubiquitin promoter-driven Renilla luciferase reporter vector pRL-Ubi to normalise the transfection efficiency. Twenty-four hours after transfection, the cells were washed with PBS and lysed in 1x passive lysis buffer (Promega, Germany) on a rocking platform for 30 minutes at room temperature. Firefly and Renilla luciferase activity were measured (substrates D-luciferin and Coelenterazine from PJK, Germany) using a Luminoscan Ascent microplate luminometer (Thermo) and a Sirius tube luminometer (Berthold), respectively. The ratios of firefly luciferase expression to Renilla luciferase expression were calculated and normalized to the TK promoter control vector, i.e. enhancer activity. For overexpression *ADCY5* cDNAs derived from SGBS total cDNA were inserted into the doxycycline-inducible Tet-On® Advanced Inducible Gene Expression System (BD Biosciences, Clontech, San Diego, CA). P-values comparing luciferase expression from risk and non-risk alleles were calculated using paired t-tests.

#### Electrophoretic mobility shift assay

EMSA was performed with Cy5-labeled oligonucleotide probes. rs56371916-flanking region oligonucleotides were commercially synthesized containing either the risk or the protective allele (Eurofins Genomics, EbersbergEurofins Genomics, Ebersberg, Germany). Cy5-labeled forward strands were annealed with non-labeled reverse strands, and the double-stranded probes were separated from single-stranded oligonucleotides on a 12% polyacrylamide gel. Complete separation was visualized by DNA shading. The efficiency of the labeling was tested by a dot plot, which confirmed that all of the primers were labeled similarly.

Primary human AMSCs were induced to differentiate into adipocytes and osteoblasts for nuclear protein harvest. Adipogenic differentiation was induced by supplementing with 66nM insulin, 100nM cortisol, 10μg/ml transferrin, 1nM triiodo-L-thyronin (T3), 2μM rosiglitazone, 25nM dexamethasone and 0.5mM IBMX. Osteogenic differentiation was induced by aMEM with 10%FCS, 1%P/S, 10mM b-glycerophosphate and 400nM hydrocortisone. Nuclear protein extracts from primary human pre-adipocytes were prepared with adapted protocols as described elsewhere(Claussnitzer et al., 2014). The supernatant was recovered and stored at −80°C. DNA-protein binding reactions were conducted in 50 mM Tris-HCl, 250 mM NaCl, 5 mM MgCl_2_, 2.5 mM EDTA, 2.5 mM DTT, 20% v/v glycerol and the appropriate concentrations of poly (dI-dC). For DNA-protein interactions, 2.5–7 μg of nuclear protein extract from the respective cell line was incubated for 10 min on ice, and Cy-5-labelled genotype-specific DNA probe was added for another 20 min. For competition experiments 50-, 100-, and 200 fold molar excess of unlabeled probe as competitor was included with the reaction prior to addition of Cy5-labeled DNA probes. Binding reactions were incubated for 20 min at 4°C. The DNA-protein complexes were resolved on a nondenaturation 5.3% polyacrylamide gel in 0.5× Tris/borate/EDTA buffer. Fluorescence was visualized with a Typhoon Trio+ imager (GE Healthcare, Munich, Munich, Germany).

**Table:** EMSA probes

rs56371916_C_for	Cy5-TGGCCCCAGAGCAGAGTGGC**C**GGCGTGAGTGAAGATGATGA-3’
rs56371916_C_rev	5’-TCATCATCTTCACTCACGCC**G**GCCACTCTGCTCTGGGGCCA-3’
rs56371916_T_for	Cy5-TGGCCCCAGAGCAGAGTGGC**T**GGCGTGAGTGAAGATGATGA-3’
rs56371916_T_rev	5’-TCATCATCTTCACTCACGCCAGCCACTCTGCTCTGGGGCCA-3’
Srebp1-competitor-for	5’-GTGGCCCCAGAGCAG**GTGGGGTGAT**GAAGATGATGAACTGG-3’
Srebp1-competitor-rev	5’-CCAGTTCATCATCTTCATCACCCCACCTGCTCTGGGGCCAC-3’

#### CRISPR/Cas9 genome editing

Plasmids: hCas9 and the gRNA cloning vector were purchased from Addgene (Plasmid ID #41815 and #41824, respectively). Genomic DNA was amplified from one rs56371916 CC allele carrier and one-TT allele carrier. Site-directed mutagenesis was performed using the Q5® Site-Directed Mutagenesis Kit (New England Biolabs) using the mutagenesis primer 5′-XXXX-3′. The guide RNAs (gRNAs) were designed using the CRISPR design online tool from the Zhang lab (http://crispr.mit.edu/). 2 guide RNAs were used: 5′ TAGAGGTCTCACCCCACTCA-3′, 5′-GAGGGGACACCTATTCCTAG-3′. For transfection, we co-transfected GFP- and hCas9- and sgRNA expression vectors, and the pMACS plasmid 4.1 (Milteny) in human AMSCs using the Amaxa-Nucleofector device (program T-030) (Lonza). We sorted cells using the MACSelect™ Transfected Cell Selection cell sorting kit (Miltenyi). Cells were sorted using the MACSelect™ Transfected Cell Selection cell sorting kit (Miltenyi). Sorted cells were cultured for 3–5 days and clones propagated from single cell were picked out. Nucleotide exchange was confirmed by Sanger sequencing and lack of random indels was confirmed by sequencing the 1000 bp genomic DNA flanking the rs56371916 targeted site. We further sequenced the top four predicted off-target sites, as computationally predicted by the CRISPR design tool (crispr.mit.edu) and the CRISPR-OFF tool ((https://rth.dk/resources/crispr/crisproff/submit), and as such we have studied all predicted off targets with a CRISPR-OFF score above 12 and predicted critical off targets based on the CRISPR-off algorithm.

#### Microarrays

Global gene expression in whole abdominal subcutaneous adipose tissue (Cohort 2) and isolated AMSCs and mature adipocytes from abdominal subcutaneous adipose tissue (Cohort 3) was measured using Illumina HumanRef-8 v.3 or HumanRef-12 v.4 BeadChip microarrays, as described previously (Dankel et al., 2010). Signal intensities were quantile normalized.

#### Lipolysis assay

Glycerol was measured in the medium after the 18-h incubation. Glycerol was measured spectrophotometrically using a glycerol 3-phosphate oxidase trinder kit (Sigma). For stimulated lipolysis measurements, 1 μmol/l isoproterenol (Sigma) was added for 1 h.

#### Palmitate oxidation assay in osteoblasts

Palmitic acid oxidation rates were determined in differentiated osteoblasts using modifications of protocols previously described(Frey et al., 2015). Fatty acid oxidation was measured in flasks with stoppers equipped with center wells. Cultures were differentiated for 0, 3, 7 days prior to analysis. The cells were rinsed with PBS and incubated with MEM supplemented with 0.5% HS and 500 μM palmitic acid for 16 h. Cells were then incubated for an additional 3 h with fresh DMEM/0.5% HS that was supplemented with [1-^14^C]palmitic acid (3.0 mCi/mmol). The oxidation reactions were terminated and CO_2_ was released from the media by the addition of 3 M perchloric acid and 1 M NaOH to the center well containing Whatman filter paper. The acidified reaction mixture was incubated overnight at 4°C and centrifuged at 4,000 rpm for 30 min before aliquots of the supernatant were counted for ^14^C-labeled acid soluble metabolites by scintillation counting of the filter paper. Each experiment was performed in triplicate and the results were normalized to total protein.

#### Alkaline Phosphatase staining

Proliferating osteoblasts show alkaline phosphatase (ALP) activity, which is greatly enhanced during *in vitro* bone formation. ALP activity is therefore a sensitive marker for osteoblast differentiation. ALP can easily be detected using BCIP (5-bromo-4-chloro-3-indolyl-phosphate) in conjunction with NBT (nitro blue tetrazolium) as a substrate, which stains cells blue-violet when ALP is present. At day 10 of differentiation, culture medium was removed and cells were carefully washed with PBS. The cellular monolayer was covered with neutral buffered formalin 10% for 60 s, then washed with 0.05% Tween 20 in PBS without Ca^2+^ or Mg^2+^ (washing buffer). Cells were incubated with BCIP/NBT substrate solution (1 tablet dissolved in 10 ml distilled water) at room temperature in the dark for 5 to 10 min, checking the staining progress every 2 to 3 min. Afterwards, the substrate solution was removed, cells were washed with washing buffer and finally kept in PBS. ALP positive cells present a dark blue-violet color, whereas AP negative cells are colorless or faintly blue.

#### Alizarin Red S staining

Osteoblasts can be induced to produce vast extracellular calcium deposits *in vitro*, a process called mineralization. Calcium deposits are an indication of successful *in vitro* bone formation and can specifically be stained bright orange-red using Alizarin Red S. The Alizarin Red S staining solution was prepared by dissolving 2 g of Alizarin Red S in 100ml distilled water and adjusting the pH to 4.1–4.3 with 0.1% NH_4_OH. After filtration, the solution was stored in the dark. At day 10 of differentiation, culture medium was removed and cells were carefully washed with PBS without Ca^2+^ or Mg^2+^. The cellular monolayer was covered with neutral buffered formalin 10% for at least 30 min, then washed with distilled water and incubated with Alizarin Red S staining solution at room temperature in the dark for 45 min. Afterwards, the substrate solution was removed, cells were washed 4 times with 1 ml distilled water and finally kept in PBS. Undifferentiated cells, without extracellular calcium deposits, are slightly red, whereas mineralized osteoblasts, with extracellular calcium deposits, are bright orange-red.

#### Seahorse XF24 Flux analyzer

Primary bone marrow stromal cells (BMSCs) were isolated from C57BL/6J male and female mice at 8–10 weeks of age as previously reported. Briefly, BMSCs were plated 2.5 × 10^4^ cells/ well in the standard 96-well Agilent sea-horse plates. BMSCs were then treated with osteogenic differentiation medium (alpha Mem, 10 FBS, 1 Pen/ Strep, supplemented with 25 μg/ mL ascorbic acid and 5 mM β-glycerolphosphate) for 0, 2, or 7 days (Guntur et al., 2018). Agilent Bioanalyzer was used to determine changes in oxygen consumption rates (OCR) and extracellular acidification rates (ECAR) and values are normalized to total protein (μg). ATP production rates were estimated using OCR and ECAR data (Mookerjee et al., 2015). We corrected ECAR by separating out contribution from CO_2_ acidification and calculated the glycolytic ATP production rate. ATP production rates from oxidative phosphorylation was then estimated from OCR by subtracting out non mitochondrial respiration and multiplying by oligomycin sensitive fraction of respiration. Total ATP production rates were obtained after adding both glycolytic and oxidative phosphorylation ATP production rates. Data are represented as % Glycolytic and oxidative phosphorylation ATP production rates. To determine the cells capacity to use free fatty acids, cells were preferentially ‘forced’ to use fatty acids (0.6 mM oleic acid) by inhibiting glucose/ pyruvate (2 μM UK5099) or glutamine (3 μM BPTES) utilization, followed by fatty acid oxidation etomoxir (4 μM etomoxir). Changes in OCR relative to substrate inhibition were then calculated.

#### ATAC-seq in primary AMSCs and immortalized AMSCs

ATAC-seq was performed by adapting the protocol from (Buenrostro et al., 2015) by adding a nuclei preparation step. Differentiating cells were lysed directly in cell culture plate at four time-points during differentiation (before adipogenesis was induced (D0, iAMSCs and AMSC), during early (D3 iAMSCs; D2 AMSC) and advanced differentiation (D6 iAMSCs and AMSC), as well as at terminal differentiation (D24 iAMSCs; D14 AMSC)). Ice-cold lysis buffer was added directly onto cells grown in a 12-well plate. Plates were incubated on ice for 10 min until cells were permeabilized and nuclei released. Cells in lysis buffer were gently scraped off the well and transferred into a chilled 1.5ml tube to create crude nuclei. Nuclei were spun down at 600 × g for 10 min at 4°C. Nuclei pellets were then re-suspended in 40μl Tagmentation DNA (TD) buffer (Nextera, FC-121-1031) and quality of nuclei assessed using trypan blue. Volume of 50,000 nuclei was determined using a hemocytometer. Transposition reaction was performed as previously described (Buenrostro et al., 2015). All tagmented DNA was PCR amplified for 8 cycles using the following PCR conditions: 72°C for 5 min, 98°C for 30 s, followed by thermocycling at 98°C for 10 s, 63°C for 30 s and 72°C for 1 min. Quality of ATAC-seq libraries was assessed using a Bioanalyzer High Sensitivity ChIP (Applied Biosystems). The profiles showed that all libraries had a mean fragment size of ∼200bp and characteristic nucleosome patterning, indicating good quality (see Figure S5). Libraries were pooled and sequenced on a HiSeq4000 Illumina, generating 50 mio reads/sample, 75-bp paired end. To reduce bias due to PCR amplification of libraries, duplicate reads were removed. Sequencing reads were aligned to hs37d5 and BWA-MEM was used for mapping. All experiments were performed in technical duplicates.

**Table:** 

Immortalized AMSCs (iAMSCs) during adipogenic differentiation		barcode	
day	rep	#	full sequence
D0	1	5	CAAGCAGAAGACGGCATACGAGATAGG AGTCCGTCTCGTGGGCTCGGAGATGT
	2	6	CAAGCAGAAGACGGCATACGAGATCATG CCTAGTCTCGTGGGCTCGGAGATGT
D3	1	7	CAAGCAGAAGACGGCATACGAGATGTAG AGAGGTCTCGTGGGCTCGGAGATGT
	2	8	CAAGCAGAAGACGGCATACGAGATCCTC TCTGGTCTCGTGGGCTCGGAGATGT
D6	1	9	CAAGCAGAAGACGGCATACGAGATAGCGT AGCGTCTCGTGGGCTCGGAGATGT
	2	10	CAAGCAGAAGACGGCATACGAGATCAGCC TCGGTCTCGTGGGCTCGGAGATGT
D24	1	13	CAAGCAGAAGACGGCATACGAGATATCAC GACGTCTCGTGGGCTCGGAGATGT
	2	14	CAAGCAGAAGACGGCATACGAGATACAG TGGTGTCTCGTGGGCTCGGAGATGT

**Table:** 

AMSC522 during Adipogenic Differentiation		Barcode	
Day	Rep	#	Full Sequence
D0	1	5	CAAGCAGAAGACGGCATACGAGATAGGA GTCCGTCTCGTGGGCTCGGAGATGT
	2	6	CAAGCAGAAGACGGCATACGAGATCATG CCTAGTCTCGTGGGCTCGGAGATGT
D2	1	8	CAAGCAGAAGACGGCATACGAGATCCTCT CTGGTCTCGTGGGCTCGGAGATGT
	2	9	CAAGCAGAAGACGGCATACGAGATAGCG TAGCGTCTCGTGGGCTCGGAGATGT
D6	1	10	CAAGCAGAAGACGGCATACGAGATCAGC CTCGGTCTCGTGGGCTCGGAGATGT
	2	11	CAAGCAGAAGACGGCATACGAGATTGCC TCTTGTCTCGTGGGCTCGGAGATGT
D14	1	12	CAAGCAGAAGACGGCATACGAGATTCCT CTACGTCTCGTGGGCTCGGAGATGT
	2	13	CAAGCAGAAGACGGCATACGAGATATCA CGACGTCTCGTGGGCTCGGAGATGT

The amount of peaks identified in the ATAC-seq data are the following:

**Table:** 

Sample:	amount of peaks:
HWAT_D0rep1	234420
HWAT_D0rep2	258532
HWAT_D3rep1	326642
HWAT_D3rep2	263894
HWAT_D6rep1	262882
HWAT_D6rep2	257710
HWAT_D24rep1	307307
HWAT_D24rep2	303605
PAC522_D0_1	393941
PAC522_D0_2	393536
PAC522_D2_1	351762
PAC522_D2_2	357997
PAC522_D6_1	424074
PAC522_D6_2	408344
PAC522_D14_1	415131
PAC522_D14_2	401583

**Figure:**
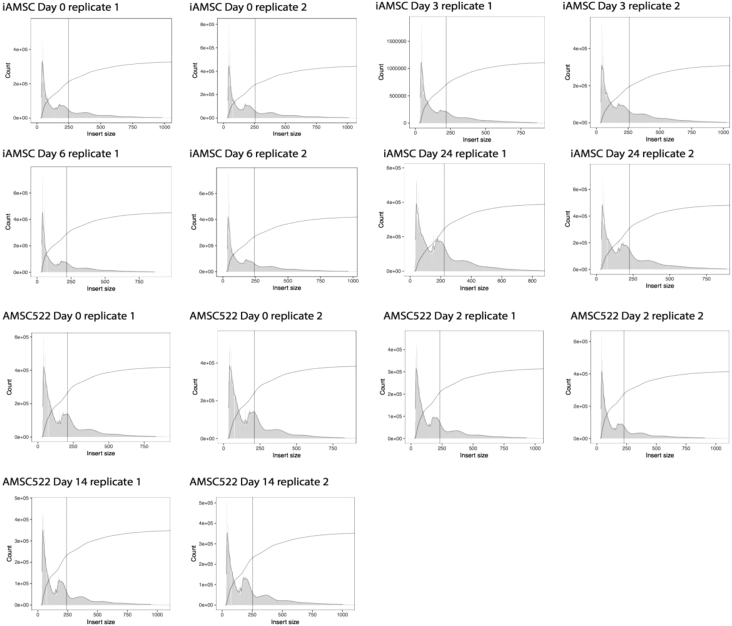

ATAC-seq fragment size and quality. Characteristic nucleosome patterning in ATAC-seq samples generated throughout adipogenic differentiation of immortalized AMSCs (Day 0, Day 3, Day 6, Day 24 of adipogenic differentiation; technical replicates were performed for each time-point) and AMSCs from donor ID 522 (Day 0, Day 2, Day 14 of adipogenic differentiation; technical replicates were performed for each time-point).

**Figure:**
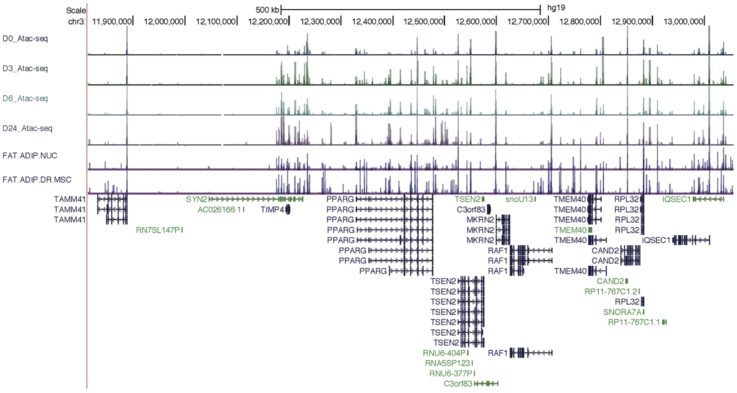

Comparison of ATAC-seq in our immortalized AMSC with DHS-seq in adipocyte nuclei from ENCODE. Histograms of peaks generated from our immortalized AMSC ATAC-seq data were visualized together with two publicly available

#### Native ChIP-seq in primary differentiating AMSCs

Native Chromatin Immunoprecipitation-Sequencing (ChIP-seq) in AMSCs was performed by adapting the protocol from (Ribarska and Gilfillan, 2018). In short, cells were lysed directly in cell culture plate at four time-points during differentiation (before adipogenesis was induced (D0), and during early (D2), mid (D6) and terminal differentiation (D14)). Ice-cold lysis buffer (containing 10 mM Tris-HCl (pH7,4), 10 mM NaCl, 3 mM MgCl2, 0.1% Igepal CA-630, supplemented with Protease Inhibitor Cocktail (Sigma P8340) and Sodium butyrate (Sigma B5887)) was added directly onto cells in culture plates. Following a 10 minutes incubation on ice, cells were scraped off plates using a cell scraper and spun down at 600 x g for 10 minutes at 4°C. The resulting nuclei pellets were resuspended in MNase Digestion buffer (50 mM Tris–HCl, pH 8.0, 1 mM CaCl2, 0.2% Triton X-100, supplemented with Protease Inhibitor Cocktail and Sodium butyrate). Nuclei were then counted using trypan blue and incubated with 2 x 10^5^ U/ml MNase-based Enzymatic Shearing Cocktail (Active Motif, 103295) for 10 min at 37°C. Subsequent steps were performed as previously described (Ribarska and Gilfillan, 2018). Native chromatin was incubated overnight at 4°C with antibodies against H3K27me3 (Diagenode, C15410069) and H3K27ac (Abcam, ab4729) using an end-to-end rotator. ChIP-seq library preparation and sequencing was performed by the Welcome Centre for Human Genetics at the University of Oxford. Libraries were pooled and sequenced on a HiSeq4000 Illumina generating approximately 50mio reads/sample, 75bp paired end. To reduce bias due to PCR amplification of libraries, duplicate reads were removed. Sequencing reads were aligned to hg19 and Bowtie 2 was used for mapping. All experiments were performed in technical duplicates. Three input control samples corresponding to the different experimental time-points (D0, D2, D6, D14) were processed and sequenced in parallel.

### Quantification and statistical analysis

#### Bivariate GWAS analyses

##### CP-ASSOC

CP-ASSOC combines GWAS summary statistics in one of two modes, that of homogeneous effects between GWAS (SHom) and that of heterogeneous effects between GWAS (SHet). These statistics are described in more detail elsewhere (Zhu et al., 2015): in brief, under the null expectation of no traits having an effect, SHom is the highest power omnibus test for any trait having an effect under an assumption of homogeneity, while SHet is a powerful statistic that does not assume homogeneity through the use of a truncated test statistic (in which only the traits with an effect above some threshold are considered, and this threshold is optimized). The SHet statistics are fit genome wide to a gamma distribution and evaluated to estimate the p-value.

We used summary statistics from a large FNBMD GWAS study performed by the GEFOS Consortium Consortium (Estrada et al., 2012);; *n*(FNBMD) = 32,961 and *n*(LSBMD) = 31,800, and from large GWAS studies from the MAGIC GWAS Consortium, glycemic trait sample (Dupuis et al., 2010; Manning et al., 2012) *n(FASTING GLUCOSE)* = 46,186, *n(FASTING INSULIN)* = 38,238, *n(HOMAIR)* = 37,037, *n(HOMAB)* = 36,466. *P*-values and minor allele frequencies from the discovery samples were included in the analyses. β coefficients and SEs from the univariate association analyses were used to perform bivariate genome-wide association analyses. We reported potential pleiotropic SNPs based on a suggestive significance level of (1) *P* ≤ 5 x 10^-06^ from the bivariate GWAS analyses; (2) the bivariate p-value divided by the univariate p-value is less than 0.05; and (3) univariate P ≤ 0.05 for both phenotypes.

##### MTAG

MTAG (Multi-Trait Analysis of GWAS) is a method to combine summary statistics from related traits in a flexible framework which takes into account the genetic correlation of the traits (Turley et al., 2017, 2018). This is particularly helpful in the case of sample overlap in individuals without population structure, as the intercept term of the genetic correlation is associated with phenotypic association while the slope is an unbiased estimator of the shared genetic effects. Under this model, there is an estimated trait covariance matrix which produces the expected effects, and various constraints on that covariance can optimize for specific assumptions of the model. Summary statistics for all traits were filtered to HapMap3 SNPs and MTAG was applied with the following command line options:

python mtag.py --ld_ref_panel ld_ref_panel/eur_w_ld_chr/ --sumstats <bone>,<glycaemic> --perfect_gencov --make_full_path --snp_name SNP --z_name Z --stream_stdout --verbose --a1_name A1 --a2_name A2 --eaf_name MAF --z_name Z --n_name N --chr_name CHR --bpos_name BP

Results with the additional option “--equal_h2” were qualitatively similar (data not shown), but given that the lack of this parameter makes fewer assumptions about the covariance structure (Turley et al., 2017), we opted for that model for downstream analysis. β coefficients and SEs from the univariate association analyses were used to perform bivariate genome-wide association analyses. We reported potential pleiotropic SNPs based onon the same criteria as CP-ASSOC above.

##### eLX

Bivariate GWAS analysis was performed using by the empirical- weighted linear-combined test statistics method (eLC) implemented in the eLX package using summary statistics from univariate GWAS meta-analyses (Chen and Hsu, 2017). The eLC directly combines correlated test statistics (or p-values) obtained from variant-phenotype association or GWAS analyses with a weighted sum of univariate test statistics. eLC maximizes the overall association signals by accounting for the correlation between phenotypes. The weighting is estimated empirically using the Monte Carlo simulation. Unfiltered summary statistics for all traits were merged by SNP name. The eLX tool was applied with the -s 1 -e <number of SNPs> -n 1 options and the dLC parametric estimate was used as the test statistic for calculating the effect, with an assumed distribution of Chi-square with two degrees of freedom (Chen and Hsu, 2017). β coefficients and SEs from the univariate association analyses were used to perform bivariate genome-wide association analyses. We reported potential pleiotropic SNPs based on the same criteria as CP-ASSOC above.

#### Chromatin state segmentation and visualization

Chromatin state segmentations were obtained from the Roadmap Epigenomics Project (Claussnitzer et al., 2014; Roadmap Epigenomics Consortium et al., 2015) and visualized in the WashU Epigenome Browser (Zhou et al., 2011). Specifically, imputed chromatin state calls from a 25-state model based on imputed datasets from 12 chromatin marks were used (Ernst and Kellis, 2012; Zhou et al., 2011). Split panels were constructed using the epigenome browser’s JSON-based configuration system. Shown are chromatin state calls across all epigenomes, as well as putative regulatory region delineations from the Roadmap Epigenomics Project.

#### H3K27me3 enrichment-based epigenome clustering

Linkage cluster trees from Roadmap (http://egg2.wustl.edu/roadmap/web_portal/epg_clustering.html) for the H3K27me3 chromatin mark were filtered to non-immortal cell lines (all consolidated epigenomes other than cancer cell lines and GM12878 from the ENCODE project). Observed H3K27me3 fold enrichment over input (“H3K27me3 signal”) was averaged over the entire risk locus for each of the epigenomes. Then, for each clade of the linkage cluster tree, a relative enrichment in H3K27me3 signal was calculated as the ratio between the average signal across epigenomes within the clade and the average signal across epigenomes outside the clade.

#### LD Score regression

Individual active histone modifications were downloaded from the list of tissue types used previously (Finucane et al., 2015 (https://data.broadinstitute.org/alkesgroup/LDSCORE/) or - for mesenchymal isolated cell types which were not included in the Finucane analysis - the corresponding files from Roadmap itself (https://egg2.wustl.edu/roadmap/web_portal/). For the qq-plots, each annotation was run through stratified LD Score regression independently, including the baseline annotations and the Roadmap “control” annotation (Finucane et al., 2018), using the default parameters. The resulting enrichments were aggregated by cell type. For the “group” annotations showing tissue class enrichment, the set of cell type group annotations that was previously reported (Finucane et al., 2015) was augmented with the aggregate signal from mesenchymal lineage cells and tissues, and each was run through the stratified LD Score regression pipeline with the default parameters, using the baseline and Roadmap “control” annotations as above.

#### Allele-specific epigenetics

Reads were mapped to the hg19 reference genome using bowtie2 (Langmead and Salzberg, 2012) and filtered to include only unambiguously mapped reads using WASP (van de Geijn et al., 2015), focusing on a 10 kb region containing the SNPs in tight LD with rs2124500 (*R*^2^ > 0.9). ATAC-seq assays were performed in technical duplicates (Pearson *R*^2^ = 0.98 day 14; *R*^2^ = 0.98 day 6; *R*^2^ = 0.98 day 2; *R*^2^ = 0.99 day 0), reads were pooled tested for allelic imbalance. The number of reads covering each haplotype (at each corresponding variant) were counted for ChIP (129 vs 147 reads) and ATAC (37 vs 19 reads) and a binomial test comparing the observed proportion of reference allele counts with the expected proportion was evaluated using a binomial test (*P* = 0.31 for ChIP, *P* = 0.02 for ATAC).

#### Repressor annotations

To identify putative stretch enhancers with cell lineage specific repression, we focused on repressed states (state 24), defined by high levels of H3 lysine 27 trimethylation (H3K27me3), associated with Polycomb repression and lower levels of promoter-associated marks (H3K4me3, H3K4me2, H3K9ac) and enhancer-associated marks (H3K4me1 and H3k27ac). To recognize master regulatory loci, we combined consecutive Polycomb-repressed elements into clusters by joining pairs of elements that were 200bp apart or less (one quarter of the median length of repressors) and evaluated total cluster length.

#### Hi-C data processing and visualization

Hi-C data from human H1-hESC derived MSC cultured cells (Dixon et al., 2015) were downloaded from SRA (SRR1030739-SRR1030744) and reprocessed using hiclib, including iterative mapping to hg19 and iterative correction (Imakaev et al., 2012), at 10kb resolution. Processing was done with both separate and combined replicates; owing to replicate similarity, the combined replicates were used for final display. Experiments from the ENCODE (DHS-seq and CTCF ChIP-seq) and Roadmap (chromatin state) projects were visualized using the WashU Epigenome Gateway (Zhou et al., 2011; interactive session 2apaIcl6nH).

#### Phylogenetic Module Complexity Analysis

We used the PMCA method described in (Claussnitzer et al., 2014) with several modifications. Briefly, multi-way multiz alignment to hg38 at UCSC (Blanchette et al., 2004) was used to define orthologous regions in 20 vertebrate species. Each region's sequence was extracted and these were aligned to each other using CLUSTALW. TF positions were selected based on matches of the given motifs to the hg38 sequence. 972 position weight matrices from the Catalog of Inferred Sequences of Binding Preferences (the Catalog of Inferred Sequences of Binding Preferences (CIS-BP)) were grouped in 192 motif matrix families using TomTom, as previously described (Maurano et al., 2015), and families were further overlapped by motif name to create a many-to-many mapping where individual TFs had multiple motifs annotated. MOODS (Korhonen et al., 2009) was used to scan a 120bp variant-flanking regions of the human reference genome (variant at mid-position) and its orthologous regions for cross-species conserved groups of transcription factor binding site motifs, so called groups of transcription factor binding site motifs, so called motif modules. A module is defined as a set of binding site motifs, whose order and distance range is conserved across species (Claussnitzer et al., 2014). The PMCA method counts instances of conserved motifs within conserved modules within the 120bp sequence context of a given variant. Enrichments of motifs in conserved modules are computed 10,000 permutations of orthologous sets. The PMCA method counts instances of conserved motifs within conserved modules within the 120bp sequence context of a given variant. The scores have a minimum of 0 (no conserved motif modules), with scores indicating the count of non-overlapping jointly conserved transcription factor binding site motifs whose relative positions within the window are conserved. Enrichments of motifs in conserved modules are computed 10,000 permutations of orthologous sets.

#### ATAC-seq-trained Basset CNN in AMSC differentiation

ATAC-seq IDR reproducible peaks for day 0, day 3, day 6, and day 24 of differentiation of immortalized AMSCs, along with peaks of DHS-seq of Osteoblasts (ENCODE file ENCFF573CUG), were collated and normalized to 20bp. A CNN Basset model (Kelley et al., 2016) was trained on genome-wide ATAC-seq data assayed in differentiating AMSCs (day 0, day 3, day 6 and day 24 of adipogenic differentiation) with two convolutional layers (512 and 128 filters; 9 and 5 filter sizes; 0.1 dropout, 1 width pooling) and two fully connected hidden layers with 128 units and 0.5 dropout, using weight normalization, a learning rate of 0.01, and momentum of 0.97. The best validation accuracy model was used for downstream analysis. Predicted relative change in chromatin accessibility (SNP accessibility difference SAD scores) between haplotype 1 and haplotype 2 in adipocytes was inferred for each SNP by centering the SNP under a 20bp window with both haplotypes and taking the difference of the predicted probabilities of an ATAC-seq peak as a measure of effect. Alleles were assigned to each SNP in high LD with rs2124500 and evaluated for predicted accessibility using Basset, in which more positive numbers indicate more predicted accessibility on the alternative allele compared to the reference allele.

*In silico* mutagenesis was performed as described in Kelley et al. (2016) to display the change in predicted accessibility for any of the four possible nucleotides. The loss score measures the largest possible decrease while the gain score measures the largest possible increase for mutation to any other non-reference nucleotide at a given position.

#### Statistics

Data was analyzed with one-way ANOVA, using Tukey’s multiple comparison test when data showed no evidence for non-Gaussian distribution. In cases where data was not normally distributed, a Kuskal-Wallis-Test was performed with a pairwise Wilcox test and corrected for multiple comparisons using the Benjamini-Hochberg procedure.

## Supplemental information

Document S1. Figures S1–S3 and Tables S2–S9

Table S1. Independent loci implicated by CP-ASSOC for association with both bone mineral density and glycemic traits and their effect on BMD, fracture, and type 2 diabetes, related to Table 1 In addition, associations discovered using the eLX and MTAG bivariate association tools. These follow the format of Table 1 and detail the bivariate association replications using eLX and MTAG. Additional details are provided for CP-ASSOC associations at all loci, and for eLX and MTAG at the *3q21.1* (*ADCY5*) locus. For each SNP, the association *Z* scores for each trait and the resulting CP-ASSOC pleiotropy p values are recorded. MTAG filters to SNPs in HapMap3 and thus only these three SNPs were tested. Bivariate SNP associations for MTAG discovered using CP-ASSOC.

Document S2. Article plus supplemental information
