# Supplementary material for: A regulatory variant at 3q21.1 confers an increased pleiotropic risk for hyperglycemia and altered bone mineral density
Source: Cell Metab. 2021 Mar 2;33(3):615–628.e13. doi: 10.1016/j.cmet.2021.01.001 (PMC7928941; doi:10.1016/j.cmet.2021.01.001)
Supplement: Document S1. Figures S1–S3 and Tables S2–S9 [file mmc1.pdf]

Cell Metabolism, Volume *33*

Supplemental information

A regulatory variant at *3q21.1* confers an increased pleiotropic risk for hyperglycemia and altered bone mineral density

Nasa Sinnott-Armstrong, Isabel S. Sousa, Samantha Laber, Elizabeth Rendina-Ruedy, Simon E. Nitter Dankel, Teresa Ferreira, Gunnar Mellgren, David Karasik, Manuel Rivas, Jonathan Pritchard, Anyonya R. Guntur, Roger D. Cox, Cecilia M. Lindgren, Hans Hauner, Richard Sallari, Clifford J. Rosen, Yi-Hsiang Hsu, Eric S. Lander, Douglas P. Kiel, and Melina Claussnitzer

A

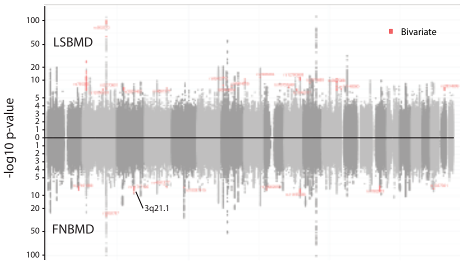

B

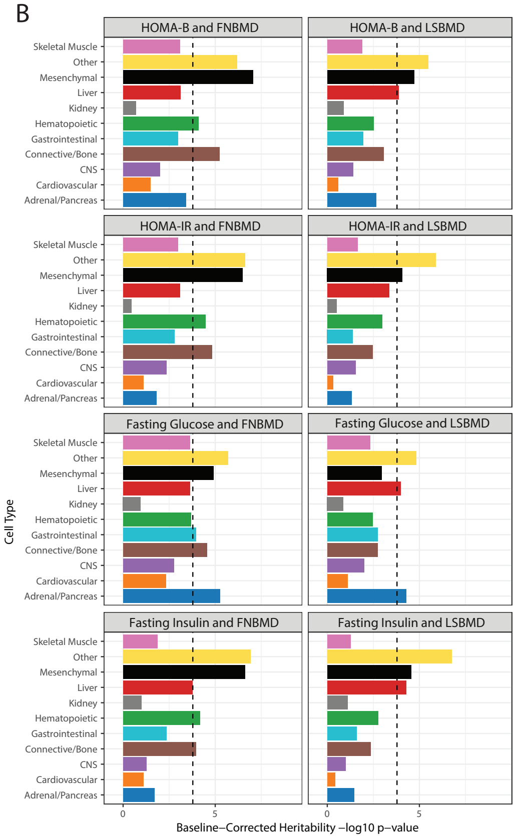

C

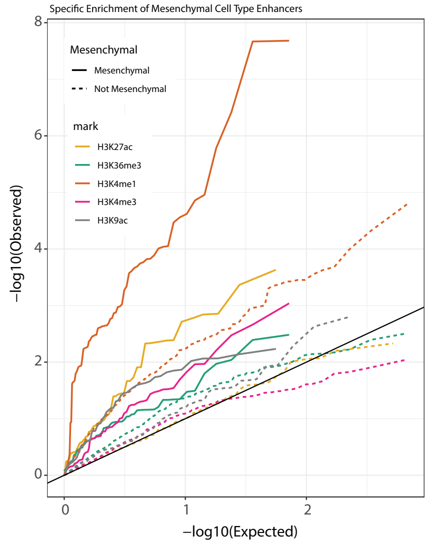

D

Emission Parameters

	H3K9me3	H3K36me3	H4K20me1	H3K79me2	H3K4me1	H3K27ac	DNase	H3K9ac	H3K4me3	H3K4me2	H2A.Z	H3K27me3
1_TssA	0.4	0.1	0.0	5.0	0.4	89.3	92.3	96.5	99.9	99.1	86.4	3.5
2_PromU	0.6	0.0	0.5	6.2	99.3	91.5	82.4	96.1	99.6	100.0	95.6	23.4
3_PromD1	0.2	1.7	7.0	98.4	60.8	99.8	92.2	100.0	100.0	100.0	93.2	6.3
4_PromD2	0.9	7.3	20.3	94.2	87.5	52.3	8.0	55.1	86.7	98.0	7.2	5.2
5_Tx5'	0.4	1.1	17.0	76.2	0.3	0.2	0.7	0.0	0.0	0.1	0.0	0.1
6_Tx	0.7	94.2	45.7	80.9	7.2	1.0	1.0	0.0	0.0	0.4	0.0	0.1
7_Tx3'	0.1	85.8	1.3	0.8	0.1	0.0	0.5	0.0	0.0	0.0	0.0	0.0
8_TxWk	0.0	2.4	0.1	1.3	0.1	0.0	0.5	0.0	0.0	0.0	0.0	0.0
9_TxReg	0.2	27.5	60.2	98.1	98.3	99.9	72.1	92.8	74.0	99.6	6.1	1.3
10_TxEnh5'	0.2	25.9	49.5	96.2	94.1	94.6	25.7	5.8	2.0	41.6	0.5	0.2
11_TxEnh3'	0.4	89.6	14.8	11.0	74.3	50.0	20.3	2.9	1.4	11.8	0.5	0.5
12_TxEnhW	0.1	9.3	48.3	95.5	76.8	3.2	6.6	0.0	0.4	18.9	0.1	0.8
13_EnhA1	0.2	4.0	1.3	5.9	99.3	99.9	83.7	95.7	38.3	95.7	43.3	0.4
14_EnhA2	0.2	0.5	0.8	2.6	97.4	97.2	59.1	7.5	9.6	96.8	29.0	0.6
15_EnhAF	0.3	0.4	0.5	2.1	97.7	94.5	31.0	3.7	1.2	2.3	6.3	0.7
16_EnhW1	0.1	0.0	0.2	0.5	91.2	16.8	39.1	3.4	15.2	73.3	46.4	0.9
17_EnhW2	0.1	0.2	0.5	1.0	75.9	0.4	13.8	0.0	0.0	1.3	0.9	0.5
18_EnhAc	0.3	0.3	0.1	1.1	4.9	64.3	19.4	0.7	0.5	3.3	1.2	0.4
19_DNase	0.1	0.0	0.1	0.0	3.4	0.3	44.7	0.0	0.0	1.4	6.2	0.1
20_ZNF/Rpts	88.9	82.0	1.0	15.9	0.5	0.1	0.6	0.2	4.7	1.4	0.0	0.1
21_Het	69.6	0.2	0.0	0.0	0.1	0.0	1.4	0.0	0.2	0.1	0.0	0.5
22_PromP	2.6	0.3	0.2	2.0	9.6	11.0	19.6	9.1	34.5	67.6	18.0	1.0
23_PromBiv	2.2	0.3	2.4	4.0	76.6	15.6	29.5	23.9	63.9	83.1	44.4	96.6
24_ReprPC	1.1	0.1	0.3	0.3	3.4	0.2	1.2	0.0	0.1	0.3	0.1	72.4
25_Quies	0.1	0.0	0.0	0.0	0.0	0.0	0.0	0.0	0.0	0.0	0.0	0.1

Median Enrichments

	Genome %	CpG.hg19	Exons.Gencodev10.hg19	Genes.Gencodev10.hg19	Introns.Gencodev10.hg19	TES.Gencodev10.hg19	TES_2kbp.Gencodev10.hg19	TSS.Gencodev10.hg19	TSS_2kbp.Gencodev10.hg19	ZNF_genes
1_TssA	0.18	97.56	10.08	1.32	0.64	3.50	2.55	96.91	9.44	3.58
2_PromU	0.41	36.50	4.75	1.12	0.84	2.94	2.37	16.12	7.18	1.86
3_PromD1	0.41	55.24	8.53	1.82	1.30	3.55	2.74	36.57	9.70	4.26
4_PromD2	0.19	2.32	3.44	1.91	1.80	3.16	2.51	2.52	7.98	4.65
5_Tx5'	2.22	0.13	0.56	1.97	2.08	0.72	0.94	0.59	1.22	2.44
6_Tx	0.70	1.34	5.15	1.96	1.72	5.95	3.83	2.52	2.84	3.25
7_Tx3'	3.48	0.95	5.57	1.93	1.65	5.93	4.34	1.99	2.58	2.76
8_TxWk	5.88	0.34	1.87	1.85	1.86	1.70	2.15	0.84	1.55	2.40
9_TxReg	0.30	2.76	4.01	1.93	1.77	4.20	2.60	3.85	5.05	1.45
10_TxEnh5'	0.38	0.36	1.81	1.96	1.97	2.00	1.54	1.48	1.96	1.41
11_TxEnh3'	0.21	1.22	7.20	1.89	1.47	7.26	4.79	2.65	3.04	1.35
12_TxEnhW	0.51	0.28	1.15	1.96	2.03	1.18	1.18	1.05	2.27	2.09
13_EnhA1	0.22	0.93	2.16	1.26	1.18	1.89	1.77	2.76	2.50	0.79
14_EnhA2	0.34	0.37	1.45	1.22	1.20	1.29	1.33	1.84	1.95	0.83
15_EnhAF	0.48	0.16	1.31	1.23	1.25	1.17	1.31	1.06	1.59	0.69
16_EnhW1	0.28	1.78	1.70	0.99	0.94	1.58	1.39	2.99	2.98	1.01
17_EnhW2	0.95	0.25	1.23	1.24	1.25	1.17	1.24	1.02	1.48	0.79
18_EnhAc	0.27	0.24	1.14	1.21	1.22	1.05	1.23	1.33	1.57	0.66
19_DNase	0.63	0.43	0.92	0.94	0.95	1.04	0.96	1.70	1.16	0.52
20_ZNF/Rpts	0.18	0.68	5.02	1.86	1.61	4.02	3.30	1.03	1.50	71.79
21_Het	0.91	0.91	1.02	0.71	0.69	0.86	0.83	0.55	0.76	7.69
22_PromP	0.20	14.16	3.01	1.22	1.09	2.22	1.79	10.48	5.02	1.51
23_PromBiv	0.25	53.53	5.88	1.31	0.95	3.71	2.62	12.96	6.81	0.72
24_ReprPC	1.32	4.88	2.02	0.99	0.92	1.74	1.69	1.69	2.75	0.47
25_Quies	78.38	0.14	0.53	0.83	0.85	0.58	0.67	0.38	0.64	0.50

E

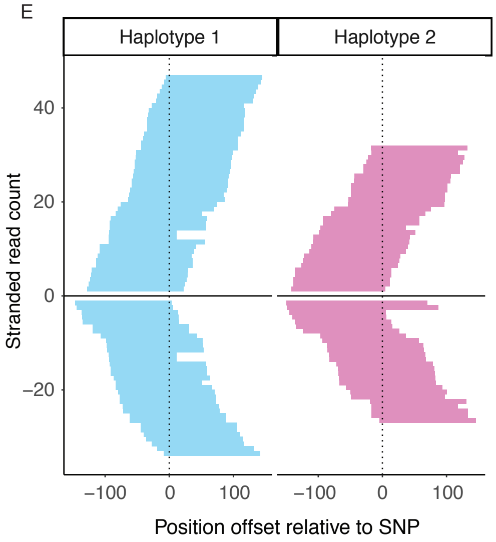

F

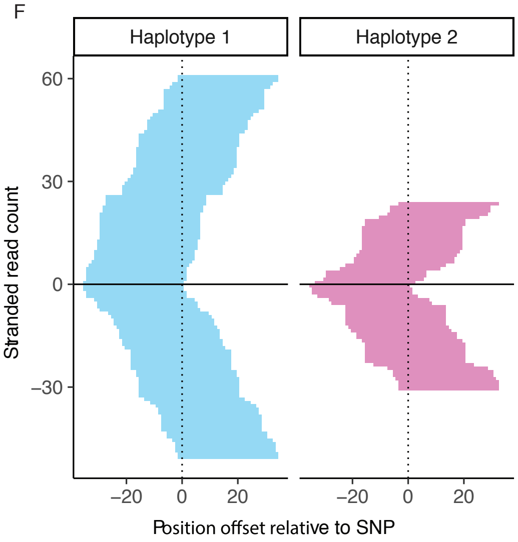

Supplementary Figure 1

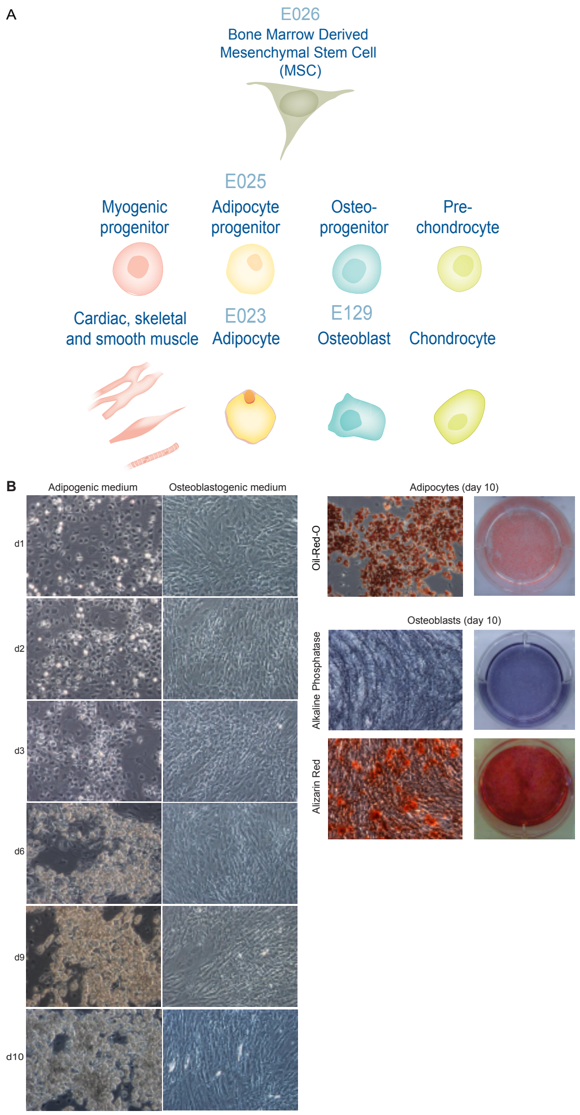

Supplementary Figure 2

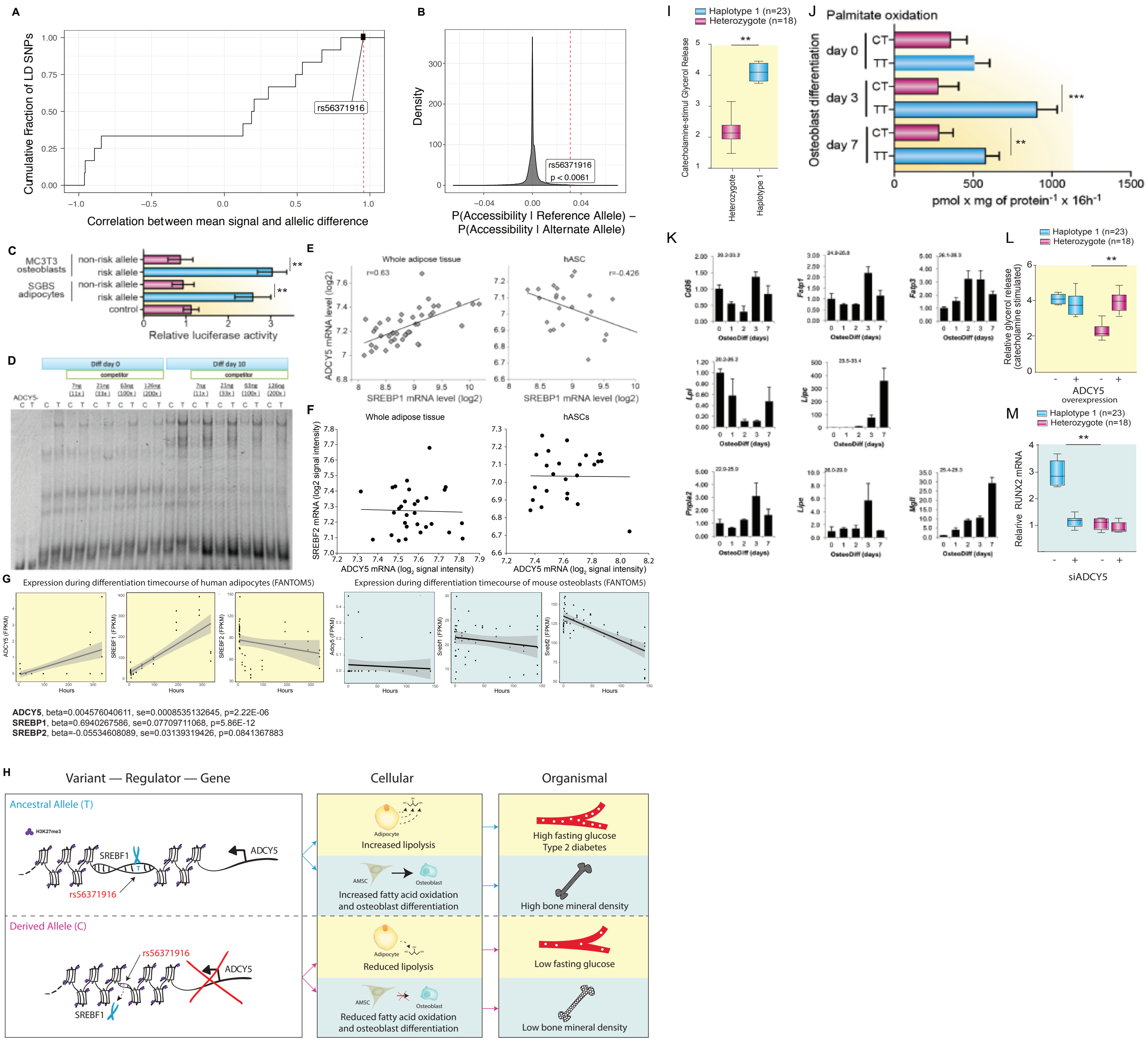

Supplementary Figure 3

Table S2. Related to Figure 2. Allelic bias through differentiation in ATAC-seq, H3K27me3 and H3K27ac ChIP-seq read counts.

Assay	Timepoint	Haplotype 1 Reads	Haplotype 1 Reads	Haplotype 2 Reads	Haplotype 2 Reads	Allelic Ratio	P-value
ATAC-seq	Day 0	25	18	9	14	1.87	0.02
ATAC-seq	Day 2	26	18	10	14	1.83	0.021
ATAC-seq	Day 14	12	25	4	15	1.95	0.02
H3K27me3 ChIP-seq	Day 0	76	60	82	52	1.01	0.95
H3K27me3 ChIP-seq	Day 2	87	59	53	68	1.21	0.46
H3K27me3 ChIP-seq	Day 14	73	73	56	84	1.04	0.31

Table S3. Related to Figure 2. Relative mRNA levels in AMSCs differentiated to osteoblasts (n=5) and adipocytes (n=5)

	**Marker Gene**	**Diff day 0**	**Diff day 6**	**Diff day 14**
Osteoblasts	*RUNX2*	1.1±0.03	1.6±0.09	*3.4±0.08*
	OCN	1.1±0.12	1.0±0.15	*2.9±0.38*
	OSX	1.0±0.01	1.3±0.07	*2.2±0.08*
Adipocytes	*CEBPA*	0.9±01	*3.1±0.03*	*3.2±0.2*
	PPARG	1.1±0.07	2.8±0.15	*3.5±0.05*
	ADIPOQ	1.0±0.2	1.3±0.07	*5.2±0.2*

Table S4. Related to Figure 3. Prioritization of the variants at the 3q21.1 locus using PMCA, Basset, and deltaSVM. Motif conservation and accessibility predictions were used. deltaSVM permutations are computed by shuffling nucleotides in the 21bp window centered on the SNP.

		PMCA conservation score		Basset accessibility score (trained on ATAC-seq data in AMSC (day 24)			deltaSVM	deltaSVM	deltaSVM
snp	r2 with rs2124500	number of jointly conserved	estimated p-value	p(accessible | haplotype 1 allele)	p(accessible | haplotype 2 allele)	p(accessible | haplotype 1 allele) - p	largest effect	ıean permutation	nax permutations
rs6794202	0.98	0	1	0.22201	0.2181	0.00391	-12.8069	874	122
rs9883204	0.97	67	0.0536	0.2132	0.21558	-0.00238	-14.3762	741	334
rs2124500	1	2	0.0013	0.2131	0.21543	0.00233	-3.04982	1205	1007
rs11720108	0.84	40	0.0002	0.18567	0.19503	-0.00936	-14.7495	743	642
rs11719201	0.84	10	0.001	0.21545	0.2121	0.00334	7.16918	1667	1032
rs35841686	0.96	24	0.0016	0.22071	0.22016	-0.00056	-3.67856	1156	1065
rs11717195	0.87	12	0.0002	0.20349	0.19531	0.00818	10.5904	1780	287
rs7614016	0.86	44	<0.0001	0.21623	0.22565	-0.00942	-10.507	875	655
rs34970607	0.96	52	<0.0001	0.20129	0.21054	-0.00925	21.3527	2019	53
rs7613951	0.86	54	<0.0001	0.19648	0.18315	0.01333	14.5136	1913	252
rs2877716	0.98	67	<0.0001	0.217	0.22147	0.00447	-8.75267	965	537
rs6798189	0.98	72	<0.0001	0.19747	0.20886	-0.01139	13.7421	1895	281
**rs56371916**	**0.98**	**189**	**<0.0001**	**0.19873**	**0.16755**	**0.03118**	17.9303	1966	70

Table S5. Related to Figure S3D. Quantification of bands from competition EMSA using ImageJ software. NE, nuclear extract.

**Well Nr.**	NE	Competitior amount	Probe	Band quantification
**1**	-	-	rs56371916 -C	
**2**	-	-	rs56371916 -T	
**3**	AMSC d0	-	rs56371916 -C	5199.865
**4**		-	rs56371916 -T	3840.087
**5**		11x	rs56371916 -C	5057.066
**6**		11x	rs56371916 -T	3722.966
**7**		33x	rs56371916 -C	4553.007
**8**		33x	rs56371916 -T	5902.572
**9**		100x	rs56371916 -C	4632.179
**10**		100x	rs56371916 -T	5491.271
**11**		200x	rs56371916 -C	2318.974
**12**		200x	rs56371916 -T	3081.894
**13**	AMSC d10	-	rs56371916 -C	7595.522
**14**		-	rs56371916 -T	10941.685
**15**		11x	rs56371916 -C	4524.936
**16**		11x	rs56371916 -T	10942.626
**17**		33x	rs56371916 -C	4423.501
**18**		33x	rs56371916 -T	6478.513
**19**		100x	rs56371916 -C	0
**20**		100x	rs56371916 -T	3936.572
**21**		200x	rs56371916 -C	0
**22**		200x	rs56371916 -T	2734.238

Table S6. Related to Figure 5. Enriched gene ontology (GO) terms for *ADCY5* co-expressed mRNAs in human adipose stromal cells (AMSC) isolated from 12 healthy non-obese patients (Pearson's $r > 0.7$).

**GO term (Pathways, humanmine.org accessed May 29 2018)**	**Enrichment p-value***	**# matches**	**% of co-expressed genes**
Co-expressed (203 recognized genes)			
Fatty acid metabolism	0.001	7	3.4
Valine, leucine and isoleucine degradation	0.027	6	3.0
Beta oxidation of octanoyl-CoA to hexanoyl-CoA	0.035	3	1.5

* P-values are Holm-Bonferroni corrected. Correlation coefficients > 0.576 were statistically significant (p-value < 0.05) (full list of genes shown in Supplemental Table 12).

Table S7. Related to Figure 5. Selected mRNAs correlated with *ADCY5* mRNA in human adipose stromal cells (AMSC) and mature adipocytes isolated from 12 healthy non-obese patients.

				AMSC		Mature adipocytes	
**Gene**	**Probe ID**	**Gene ID**	**Definition**	**Pearson's r**	**P-value**	**Pearson's r**	**P-value**
Co-expressed with ADCY5 (Fatty acid metabolism)							
ACADM	ILMN_2053679	34	acyl-Coenzyme A dehydrogenase, C-4 to C-12 straight chain (ACADM), nuclear gene	0.764	0.003	-0.092	0.776
ACAT1	ILMN_1800008	38	acetyl-Coenzyme A acetyltransferase 1 (acetoacetyl Coenzyme A thiolase) (ACAT1), r	0.756	0.004	0.380	0.225
ADH1A	ILMN_1764309	124	alcohol dehydrogenase 1A (class I), alpha polypeptide (ADH1A)	0.862	0.000	0.096	0.769
ADH1B	ILMN_1811598	125	alcohol dehydrogenase IB (class I), beta polypeptide (ADH1B)	0.718	0.009	0.208	0.518
CPT2	ILMN_1678579	1376	carnitine palmitoyltransferase II (CPT2), nuclear gene encoding mitochondrial protein	0.761	0.005	0.061	0.843
HADH	ILMN_1719906	3033	hydroxyacyl-Coenzyme A dehydrogenase (HADH), nuclear gene encoding mitochondr	0.796	0.002	0.137	0.672
HADHB	ILMN_2197846	3032	hydroxyacyl-Coenzyme A dehydrogenase/3-ketoacyl-Coenzyme A thiolase/enoyl-Coer	0.707	0.010	-0.211	0.515
Fatty acid metabolism (marker genes)							
ACACA	ILMN_1772123	31	acetyl-Coenzyme A carboxylase alpha (ACACA), transcript variant 2	0.426	0.163	0.044	0.891
ACACB	ILMN_1763852	32	acetyl-Coenzyme A carboxylase beta (ACACB)	0.475	0.116	0.597	0.041
ADFP/PLIN2	ILMN_1801077	123	adipose differentiation-related protein (ADFP)	0.207	0.543	0.111	0.187
ADIPOQ	ILMN_1775045	9370	adiponectin, C1Q and collagen domain containing (ADIPOQ)	0.677	0.017	0.497	0.102
CD36	ILMN_1665132	948	CD36 molecule (thrombospondin receptor) (CD36), transcript variant 2	0.444	0.150	0.009	0.572
CEBPA	ILMN_1715715	1050	CCAAT/enhancer binding protein (C/EBP), alpha (CEBPA)	0.571	0.051	0.012	0.973
LIPE	ILMN_1670693	3991	lipase, hormone-sensitive (LIPE)	0.556	0.062	0.234	0.463
MGLL	ILMN_1707310	11343	monoglyceride lipase (MGLL), transcript variant 2	0.275	0.404	0.339	0.281
PLIN	ILMN_1665562	5346	perilipin (PLIN)	0.607	0.037	0.557	0.062
PNPLA2	ILMN_1787923	57104	patatin-like phospholipase domain containing 2 (PNPLA2)	0.321	0.306	0.421	0.171
PPARG	ILMN_1800225	5468	peroxisome proliferator-activated receptor gamma (PPARG), transcript variant 2	0.694	0.012	0.049	0.880
SLC27A1	ILMN_1787718	376497	solute carrier family 27 (fatty acid transporter), member 1 (SLC27A1)	0.143	0.675	0.513	0.089
Osteoblast differentiation and function (marker genes)							
KLF15	ILMN_1683133	28999	Kruppel-like factor 15 (KLF15)	0.761	0.004	0.179	0.581
LIF	ILMN_1738725	3976	leukemia inhibitory factor (cholinergic differentiation factor) (LIF)	-0.854	0.0003	-0.235	0.452
OCX/SP7	ILMN_1689461	121340	Sp7 transcription factor (SP7)	-0.089	0.811	0.229	0.482
OSN/BGLAP	ILMN_1755818	632	bone gamma-carboxyglutamate (gla) protein (osteocalcin) (BGLAP)	0.070	0.829	-0.156	0.628
RUNX2	ILMN_1716651	860	runt-related transcription factor 2 (RUNX2), transcript variant 2	-0.487	0.117	0.635	0.027
ZNF26	ILMN_1691798	7574	zinc finger protein 26 (ZNF26)	-0.713	0.009	-0.062	0.837
ZNF74	ILMN_2383871	7625	zinc finger protein 74 (ZNF74)	0.640	0.026	-0.407	0.189
ZNF133	ILMN_2174081	7692	zinc finger protein 133 (ZNF133)	0.750	0.005	0.336	0.289
ZNF319	ILMN_1711361	57567	zinc finger protein 319 (ZNF319)	0.799	0.002	0.149	0.643
ZNF485	ILMN_1664034	220992	zinc finger protein 485 (ZNF485)	0.710	0.010	0.013	0.981

Correlation coefficients > 0.576 were statistically significant (p-value < 0.05). Correlations were calculated based on log2-transformed mRNA expression values.

Table S8. Related to Figure 5. Fold change and significance (p-value) of expression changes in primary human osteoblasts and adipocytes (haplotype 1 and haplotype 2). p-values were calculated by Mann Whitney U test.

		**Homozygous/ Heterozygous**	
	**Gene**	**Fold change (±SE)**	**p-value**
Osteoblasts	*RUNX2*	1.9±0.42	0.06
	OCN	1.4+0.32	0.04
	OSX	1.2+0.27	0.001
	ACCB	1.1+0.13	0.04
	ACAT1	1.5+0.31	0.05
	CPT1	1.7+0.22	0.001
Adipocytes	*ATGL*	1.3±0.35	0.02
	LIPE	2.1+0.56	0.01
	PLIN2	1.4+0.25	0.05

Table S9. Related to Figure 5. CRISPR based single-nucleotide editing reverses osteoblast differentiation and adipocyte lipid metabolism marker genes, confirming pleiotropy.

Relative mRNA levels in osteoblasts (ratio TT/CC)

**Gene**	**Fold change**	**p-value**	**Fold change (±S**	**p-value**
ADCY5	1.6+0.4	0.0286	1.1+0.2	n.s.

Relative AP activity levels in osteoblasts (ratio TT/CC)

			**Diff day 14**	
			**Fold change**	**p-value**
			1.6+0.2	0.01

Relative mRNA levels in adipocytes (ratio CC/TT)

**Gene**	**Fold change**	**p-value**	**Fold change**	**p-value**
ADCY5	0.7+0.3	0.0286	1.03+0.3	n.s.

Relative mRNA levels in adipocytes (ratio TT/CC)

	**Diff day 0**		**Diff day 14**	
**Gene**	**Fold change**	**p-value**	**Fold change**	**p-value**
ATGL	1.3+0.4	0.02	1.5+0.9	0.07
HSL	1.8+0.63	0.09	1.8+0.4	0.03
PLIN2	1.9+0.42	0.05	1.9+1.2	0.05

Figure S1. Manhattan plot of genome-wide association results for bone density and glycemic traits using CP-ASSOC (Related to Figure 1).

Panel A. Bivariate genetic associations for two bone traits LSBMD and FNBMD and four glycemic traits (HOMA-IR, HOMA-B, fasting glucose levels, and fasting insulin levels). Genetic loci passing bivariate criteria are highlighted in red.

Panel B. Stratified LD score regression analysis (Finucane et al., 2015) across the entire bivariate GWAS for all FNBMD, LSBMD, FG, FI, HOMAIR and HOMAB pairs using active histone modification genome-wide ChIP-seq data from diverse cell types and tissues. Cutoff for Bonferroni significance (-log10(P) = 3.25) is indicated by black dotted lines.

Panel C. Stratified LD score regression analysis (Finucane et al., 2015) for mesenchymal versus non-mesenchymal cell type groups for diverse histone marks.

Panel D. Annotation panel and color key for the 25 chromatin state model (Roadmap Epigenomics Consortium et al., 2015). Rows represent states and columns are emission parameters (left table) and enrichments of relevant genomic annotations (right panel).

Panel E. Allelic imbalance analysis of the *3p21.1* risk locus for chromatin accessibility by ATAC-seq in heterozygous AMSCs from two individuals comparing haplotype 1 (blue) and haplotype 2 (pink). Each panel depicts ATAC-seq read counts separated by strand above/below the midpoint.

Panel F. Allelic imbalance analysis of the *3p21.1* risk locus for chromatin accessibility by DHS-seq in heterozygous skeletal muscle-derived MSCs (Maurano et al., 2015) comparing haplotype 1 (blue) and haplotype 2 (pink). Each panel depicts DHS-seq read counts separated by strand above/below the midpoint.

Figure S2. AMSC-derived adipocytes and osteoblast differentiation model system (Related to Figure 2).

Panel A. Lineage relationships of MSCs and MSC-derived lineages. Epigenome identity (EID) numbers from the Roadmap Epigenomics (Roadmap Epigenomics Consortium *et al.*, 2015) are indicated.

Panel B. Microscopic visualization of morphological and cellular changes of AMSCs during adipocyte and osteoblast from bright field microscopy, Oil-Red-O lipid staining (adipocytes) and Alkaline Phosphatase and Alizarin Red (osteoblasts). Representative images are shown from three replicates.

Figure S3. rs56371916 – SREBP1 – ADCY5 regulatory circuitry and its cellular mechanisms in adipocytes and osteoblasts (Related to Figure 3 to 5).

Panel A. Correlation of predicted SNP accessibility difference from the CNN Basset and chromatin accessibility for variants at the *3p21.1* locus. rs56371916: r = 0.957, P = 0.04.

Panel B. Distribution of CNN predicted SNP accessibility for 29,472 trait-associated SNPs in the GWAS catalog. Empirical P (rs56371916) = 0.0061.

Panel C. rs56371916 allele-specific luciferase assays for 1kb centered on rs56371916 in adipocytes and osteoblasts. Mean + SD $^{**}P < 0.01$.

Panel D. Competition EMSA assays using adipocyte nuclear extract and 11-, 33-, 100-, and 200-molar excess of unlabeled probes. Representative blot from three replicates is shown.

Panel E. Correlation of *ADCY5* mRNA with *SREBP1* mRNA in human subcutaneous adipose tissue from 30 (Cohort 2) and subcutaneous AMSCs from 24 individuals (Cohort 3), measured by Illumina microarrays. Pearson's r (whole tissue) = 0.63, P = 7 x 10-06, r (AMSC) = - 0.426, P = 0.038.

Panel F. Correlation of *ADCY5* mRNA with *SREBP2* mRNA in human subcutaneous adipose tissue from 312_270 (Cohort 2) and subcutaneous AMSCs from 24 lean individuals (Cohort 3), measured by Illumina microarrays.

Panel G. Quantification of gene expression for *ADCY5, SREBP1*, and *SREBP2* in differentiating human adipocytes (yellow background) and differentiating mouse osteoblasts (blue background) from the FANTOM5 database.

Panel H. Schematic regulatory model of the *3p21.1* risk locus.

Panel I. Catecholamine-stimulated glycerol release in differentiated adipocytes from 23 homozygotes and 18 heterozygotes for haplotype 1 (Cohort 1). Mean + SD $^{**}P < 0.01$.

Panel J. Oxidation of [14C]-palmitate to 14CO2 at different stages of osteoblast differentiation (day 0, day 3, and day 7). Mean + SD $^{**}P < 0.01$; $^{***}P < 0.001$; n = 4 (haplotypes 1/2) and n= 4 (haplotypes 1/1), Cohort 4.

Panel K. Quantification of gene expression of marker genes involved in fatty acid transport and lipid oxidation in murine bone marrow stromal cells (BMSCs) differentiated to osteoblasts (day 0, 1, 2, 3, 7).

Panel L. Catecholamine-stimulated glycerol release upon doxycycline-induced overexpression of ADCY5 in adipocytes from qPCR. Mean + SD $^{**}P < 0.01$; n = 4 (haplotypes 1/2) and n= 4 (haplotypes 1/1), Cohort 4.

Panel M. Quantification of RUNX2 gene expression from qPCR upon siRNA knock-down of ADCY5 (56% knock-down efficiency). Mean + SD $^{**}P < 0.01$; n = 18 (haplotypes 1/2) and n= 23 (haplotypes 1/1), Cohort 1.
